# Structural basis of astrocytic Ca^2+^ signals at tripartite synapses

**DOI:** 10.1038/s41467-020-15648-4

**Published:** 2020-04-20

**Authors:** Misa Arizono, V. V. G. Krishna Inavalli, Aude Panatier, Thomas Pfeiffer, Julie Angibaud, Florian Levet, Mirelle J. T. Ter Veer, Jillian Stobart, Luigi Bellocchio, Katsuhiko Mikoshiba, Giovanni Marsicano, Bruno Weber, Stéphane H. R. Oliet, U. Valentin Nägerl

**Affiliations:** 1 University of Bordeaux, Bordeaux, France; 2 Interdisciplinary Institute for Neuroscience, CNRS UMR 5297, Bordeaux, France; 3 NeuroCentre Magendie, Inserm U1215, Bordeaux, France; 4 Bordeaux Imaging Center, University of Bordeaux, Bordeaux, France; 5 Bordeaux Imaging Center, CNRS UMS 3420, Bordeaux, France; 6 Bordeaux Imaging Center, INSERM US04, Bordeaux, France; 7 University of Zurich, Institute of Pharmacology & Toxicology, Zürich, Switzerland; 8 ShanghaiTech University, Shanghai, 201210 China

**Keywords:** Super-resolution microscopy, Cellular neuroscience, Astrocyte

## Abstract

Astrocytic Ca^2+^ signals can be fast and local, supporting the idea that astrocytes have the ability to regulate single synapses. However, the anatomical basis of such specific signaling remains unclear, owing to difficulties in resolving the spongiform domain of astrocytes where most tripartite synapses are located. Using 3D-STED microscopy in living organotypic brain slices, we imaged the spongiform domain of astrocytes and observed a reticular meshwork of nodes and shafts that often formed loop-like structures. These anatomical features were also observed in acute hippocampal slices and in barrel cortex in vivo. The majority of dendritic spines were contacted by nodes and their sizes were correlated. FRAP experiments and Ca^2+^ imaging showed that nodes were biochemical compartments and Ca^2+^ microdomains. Mapping astrocytic Ca^2+^ signals onto STED images of nodes and dendritic spines showed they were associated with individual synapses. Here, we report on the nanoscale organization of astrocytes, identifying nodes as a functional astrocytic component of tripartite synapses that may enable synapse-specific communication between neurons and astrocytes.

## Abstract (web summary)

Astrocytic Ca^2+^ signals can be fast and local, supporting the idea that astrocytes have the ability to regulate single synapses. Here, the authors report the organization of astrocytes at nanoscale level and identify nodes as a functional astrocytic component of tripartite synapses.

## Introduction

The concept of the tripartite synapse posits that the flow of information at synapses is a result of dynamic signaling between pre- and post-synaptic neurons as well as astrocytes^1^. It assigns astrocytes an active and pivotal role in information processing in the brain, beyond their multiple homeostatic functions.

The thin processes of protoplasmic astrocytes in the cortex and hippocampus, which characteristically traverse the neuropil at very high density, come into close physical contact with many synapses, appearing to wrap around them in electron microscopy images^2^. In addition, neurotransmitters released from presynaptic nerve terminals not only bind to postsynaptic receptors, but are also detected by astrocytes, leading to Ca^2+^ elevations and release of neuroactive substances, such as glutamate, D-serine, and ATP^3^. In turn, these gliotransmitters can influence synaptic function, including basal synaptic transmission^4^^,^^5^ and long-term synaptic plasticity^6–8^.

While astrocytic Ca^2+^ elevations have long been thought of only as slow and spatially spread out, recent studies suggest that the situation in astrocytic processes may actually not be so different from neurons, which exhibit fast Ca^2+^ signals in microdomains^4,5,9–16^.

Neurons have a very elaborate morphology, which helps them spatially compartmentalize diffusible signaling molecules or ions like Ca^2+^. A prime example of structural compartmentalization are mushroom-shaped dendritic spines where the spine neck reduces biochemical cross talk between neighboring spine heads, enabling synapse-specific changes in synaptic strength^17^. In the same vein, astrocytes also have an extremely intricate morphology, which could provide the structural basis for compartmentalized intracellular signaling^18^.

Local Ca^2+^ signals have been reported in morphological enlargements on major branches of astrocytes^5^ and on processes of Bergmann glial cells^19^. However, we know very little about the structural basis of Ca^2+^ signals in the spongiform domain of astrocytes, where astrocytic processes come into contact with thousands of neuronal synapses they potentially regulate.

The lack of information stems largely from the difficulty of visualizing the spongiform domain with conventional light microscopy, which does not have enough spatial resolution for this^20,21^. While EM has enough resolution, it can only provide snapshots and may give a distorted view of the morphological relationships of the tripartite synapse in the case of chemically fixed brain tissue^22^.

We overcame these problems by applying live-cell 3D-STED microscopy^23^ to resolve the morphological details of astrocytes and to see how they contact dendritic spines in living brain tissue.

We observed looped structures in the images of spongiform domain of astrocytes, which seemed to form continuous genuine rings, according to 3D reconstructions.

We observed that the majority of contacts with dendritic spines were formed by bulbous enlargements, or nodes, which were assembled along thin astrocytic processes, or shafts. Most nodes were actually branch points that gave rise to multiple shafts. We also observed this anatomical organization in acute hippocampal slices and in barrel cortex in vivo. Large spines tended to be associated with large nodes, forming stable contacts.

FRAP (fluorescence recovery after photobleaching) experiments and confocal Ca^2+^ imaging showed that nodes were diffusionally isolated compartments that hosted highly localized spontaneous Ca^2+^ transients. Finally, mapping the Ca^2+^ transients onto STED images of dendrites, these localized Ca^2+^ signals occurred in nodes that were in contact with dendritic spines.

Taken together, our study sheds new light on the micro-anatomical architecture and Ca^2+^ activity of astrocytes in the spongiform domain, identifying astrocytic nodes as the morphological structures that host Ca^2+^ transients at individual tripartite synapses.

## Results

### The spongiform domain features a meshwork of nodes and shafts

To visualize astrocytic morphology in the spongiform domain, we used a custom-built 3D-STED microscope with a spatial resolution of around 50 nm in x–y and 200 nm in z. We labeled the astrocytes with a cytosolic fluorescent marker protein (ZsGreen) in living organotypic mouse brain slices. This sample preparation maintains an organotypic organization, yet provides nearly ideal conditions in terms of optical access and sample stability, which is critical for super-resolution imaging^23^.

**Fig. 1:**
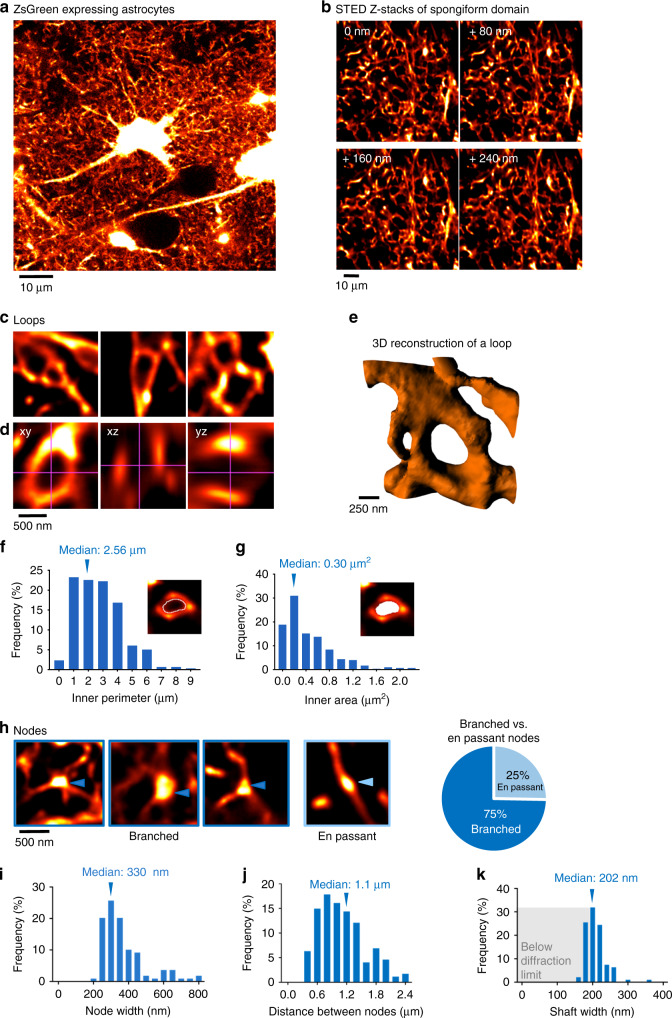
Spongiform domain is formed by meshwork of nodes and shafts, and features loops. **a** Confocal overview image of astrocytes expressing ZsGreen. **b** Z-stack STED images of spongiform domain showing an elaborate reticular meshwork. **c** Loop-like structures observed in the spongiform domain. **d** Orthogonal views of a loop-like structure. **e** 3D view of the loop-like structure shown in **d**. **f** Frequency distribution of loop inner perimeter (*n* = 297 from 4 slices). **g** Frequency distribution of loop inner area (*n* = 297 from 4 slices). **h** Nodes at branch points (branched) and along the shaft (en passant) (left) and the percentage of respective structures (right). Nodes frequently form branch points (*n* = 261 nodes from 14 slices). **i** Frequency distribution of node width (*n* = 109 nodes from 14 slices). **j** Frequency distribution of shaft length connecting two neighbor nodes. Nodes were closely spaced (*n* = 174 shafts from 11 slices). **k** Distribution of shaft width. Shaft width was frequently below the diffraction limit of conventional light microscopy (<200 nm; gray box), making the use of super-resolution approach necessary (*n* = 94 shafts from 14 slices). (See also Supplementary Figs. 1 and 2).

Our approach revealed a complex three-dimensional topology, resembling a reticular meshwork of dividing and merging astrocytic processes (Fig. 1a, b). In individual sections, we often saw circular structures (median inner perimeter: 2.56 µm; median inner area: 0.30 µm^2^) (Fig. 1c, f, g). Based on orthogonal views and 3D volume reconstructions, some of the loops appeared to be genuine closed rings formed by reconnecting astrocytic processes (Fig. 1d, e, Supplementary Movie 1).

The meshwork featured closely spaced bulbous node-like enlargements (median distance: 1.1 µm; median width: 330 nm) (Fig. 1h–j) that frequently formed branch points (75%) from which three or more thinner connecting shaft-like processes emerged (median width: 202 nm) (Fig. 1k). Given that these sizes are around the diffraction barrier of light microscopy, the use of super-resolution microscopy was necessary to be able to make accurate geometric measurements.

While the 3D-STED approach was much better at revealing morphological details than confocal microscopy (Supplementary Fig. 1), this is not to say that it could reveal all of them equally well. For instance, sheet-like structures reported in the EM literature remained relatively elusive, even when using a membrane-targeted fluorescent label (Lck-clover; Supplementary Fig. 2a–c). Hence, we focused our attention on the structures we could reliably detect with the cytosolic label.

The anatomical organization of nodes and shafts was equally discernible in regions proximal and distal to the cell body, and nodes were found at similar densities in both regions (Supplementary Fig. 2d–f). Moreover, we saw no evidence for distal tapering of astrocytic processes in the spongiform domain (Supplementary Fig. 2e), unlike the situation for neuronal dendrites, which generally become thinner towards their distal tips. In fact, hyper-thin shafts could be seen to emerge directly from the cell body and major branches, which is in line with an EM study^11^.

**Fig. 2:**
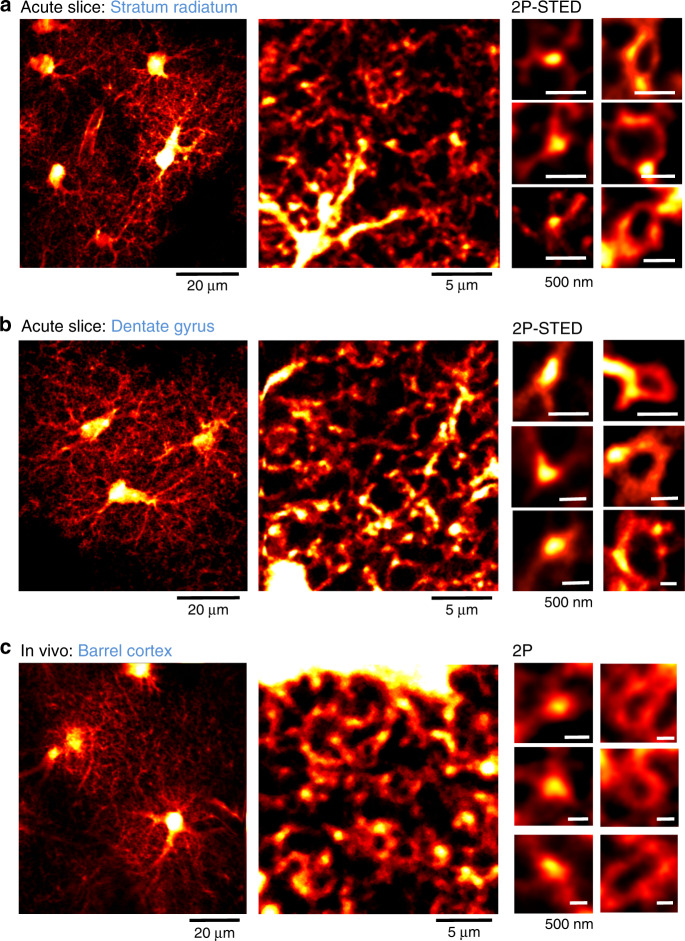
Novel astrocytic structures also exist in different experimental preparations and brain regions. Left: 2P overview image of astrocytes expressing Clover in stratum radiatum (**a**), dentate gyrus (**b**) from acute slices (*n* = 4 slices), and in barrel cortex in vivo (**c**) (*n* = 5 animals). Middle: 2P image of spongiform domain displaying a reticular meshwork in stratum radiatum (**a**), dentate gyrus (**b**) from acute slices (*n* = 4 slices), and in barrel cortex in vivo (**c**) (*n* = 5 animals). Right: 2P-STED (**a**, **b**) or 2P (**c**) images of loop-like structures (left) and nodes (right) in stratum radiatum (**a**), dentate gyrus (**b**) from acute slices (*n* = 4 slices), and in barrel cortex in vivo (**c**) (*n* = 5 animals).

We also observed these structures (loops, nodes, and shafts) in different brain regions (CA1 stratum radiatum, dentate gyrus, and barrel cortex), more physiological preparations (acute hippocampal slices and in vivo) and with a different fluorescent protein (Clover), indicating they are a general anatomical feature of astrocytes in the mouse brain and not just particularities or artifacts of organotypic slices or the labeling method (Fig. 2).

### The majority of perisynaptic astrocytic structures are nodes

**Fig. 3:**
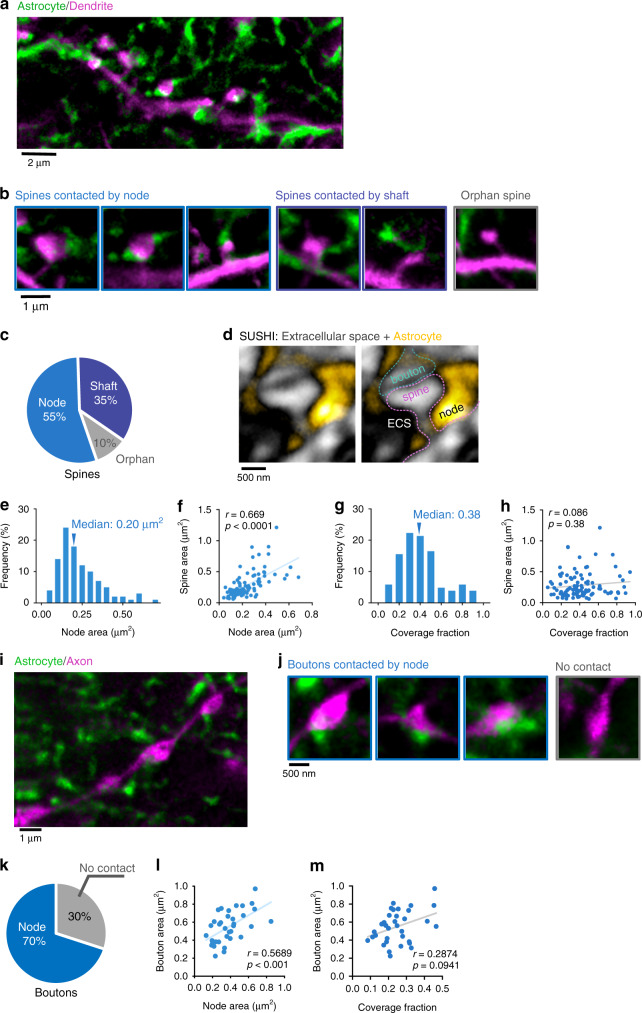
Majority of spines are contacted by nodes. **a** Two-color STED image of morphological interaction between astrocytic spongiform domain (green) and a dendrite (magenta). **b** Images of spines contacted by nodes, shafts, and spines lacking an astrocytic structure in their vicinity. **c** Percentage of spines contacted by nodes, shafts, and spines lacking an astrocytic structure in their vicinity. The majority of spines are contacted by at least one node (*n* = 188 spines from 21 slices). **d** Image of extracellular space (black) surrounding a synapse (gray) and astrocyte (yellow). Right image indicates the putative identity of the synaptic components. **e** Frequency distribution of node area. Nodes were highly variable in size (*n* = 103 nodes from 21 slices). **f** Correlation between node area and spine head area. Node area was strongly positively correlated with spine head area (*n* = 103 structures from 21 slices; Spearman *r* = 0.669, ****p (two-tailed) < 0.0001). **g** Frequency distribution of fraction of spine coverage by astrocytic node, showing that spine surfaces were mostly node-free (*n* = 103 spines from 21 slices). **h** Correlation between coverage fraction and spine head area. Large spines had similar coverage fractions as small spines (*n* = 103 spines from 21 slices; Spearman *r* = 0.086, *p* (two-tailed) = 0.38). **i** Two-color STED image of morphological interaction between astrocytic spongiform domain (green) and an axon (magenta). **j** Images of boutons contacted by nodes and boutons lacking an astrocytic structure in their vicinity. **k** Percentage of boutons contacted by nodes and boutons lacking an astrocytic structure in their vicinity. Node-contacted boutons account for the majority of boutons (*n* = 35 boutons from 5 slices). **l** Correlation between node area and bouton head area. Bouton area was positively correlated with node area (*n* = 35 structures from 7 slices; Spearman *r* = 0.5689, ***p (two-tailed) = 0.0004). **m** Correlation between fraction of bouton coverage by astrocytic node and bouton area. Large boutons had similar coverage fractions as small boutons (*n* = 35 boutons from 5 slices; Spearman *r* = 0.2874, *p* (two-tailed) = 0.0941).

Given that nodes and shafts are ubiquitous structures in the spongiform domain where most of the synaptic contacts occur, we wondered how these astrocytic structures interact with excitatory synapses formed by dendritic spines in the spongiform domain. To address this question, we performed two-color STED imaging of co-labeled astrocytes and neurons, and analyzed their spatial relationships (Fig. 3a).

While around 55% of spines were contacted by at least one node, the remainder were either contacted by shafts (35%) or did not have any astrocytic structure in their vicinity (10%) (Fig. 3b, c). Applying the recently developed super-resolution shadow imaging (SUSHI) technique^23^, we observed a relatively spacious distribution of the extracellular space around dendritic spines (Fig. 3d). This is very different from EM images based on chemical fixation, where hyper-thin astrocytic processes can be seen to wrap closely around synapses^2,24^, leaving virtually no extracellular space.

### Astrocytic nodes and dendritic spines are correlated in size

Given that astrocytic nodes are highly variable in size (0.07–0.7 µm^2^; median area = 0.20 µm^2^; Fig. 3e), we examined whether they correlated with the size of the spine they contacted. Indeed, we observed a strong correlation (*r* = 0.669, *p* < 0.0001, Fig. 3f). In addition, spines with two nodes tended to be larger than those with one node (Supplementary Fig. 3a–d), suggesting that spines and nodes are linked functionally.

To examine these morphological interactions in greater detail, we quantified the coverage fraction (defined as the fractional perimeter of the spine head that is in contact with a node). The median value of the coverage fraction was 0.38, indicating that spine surfaces were mostly node-free (Fig. 3g). Furthermore, spine size did not correlate with coverage fraction, i.e. large spines had similar coverage fractions as small spines (Fig. 3h).

We observed a similar structural relationship between nodes and presynaptic boutons. Most boutons (70%) were contacted by nodes, correlated in size with them and had similar coverage fractions (Fig. 3i–m), similar to the situation with spines.

### Node-spine contacts are largely stable

**Fig. 4:**
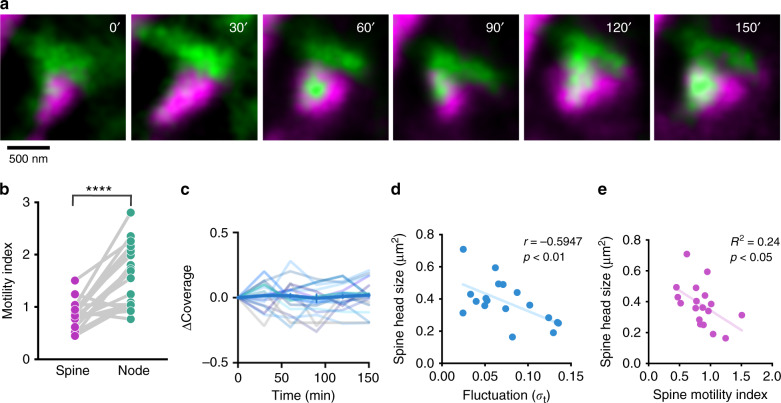
Node-spine contacts are largely stable. **a** Time-lapse STED imaging of a spine (magenta) and a node (green) over 150 min acquired every 30 min. While contacting node can move on the spine surface, the overall contact is maintained. **b** Comparison between spine motility index and contacting nodes. Motility index of the node was significantly higher than that of spines (*n* = 18 structures from 5 slices; Paired *t*-test, ****p (two-tailed) < 0.0001). **c** Changes of coverage state over time normalized to the coverage state at 0 min (*n* = 18 spines from 5 slices). **d** Correlation between fluctuation (the temporal variance of coverage fraction) and spine head area (*n* = 18 spines from 5 slices; Spearman *r* = −0.5947, *p* (two-tailed) = 0.0092. Large node/large spine contact was more stable. **e** Correlation between spine head size and spine motility index. Large spines were less motile than small spines (*n* = 18 spines from 5 slices; Pearson *R*^2^ = 0.24, *p* (two-tailed) = 0.039).

Next, we assessed the stability of the node-spine contacts by two-color time-lapse STED imaging. While nodes were more motile than spines, most contacts were maintained for at least 2.5 h (Fig. 4a, b). We observed only modest fluctuations in the coverage fraction, reflecting subtle changes in the shape and relative position of the nodes and spines (Fig. 4c). We observed a negative correlation between fluctuation, i.e. the temporal variance of coverage fraction, and spine size, suggesting that large spines form more stable contacts with nodes (Fig. 4d), which can be explained as large spines tend to be less motile (Fig. 4e). By comparison, microglia are known to form only short-lived (~minutes) contacts with spines in the somatosensory cortex in vivo^25^ and hippocampal slices^26^.

### Biochemical compartmentalization by nodes

**Fig. 5:**
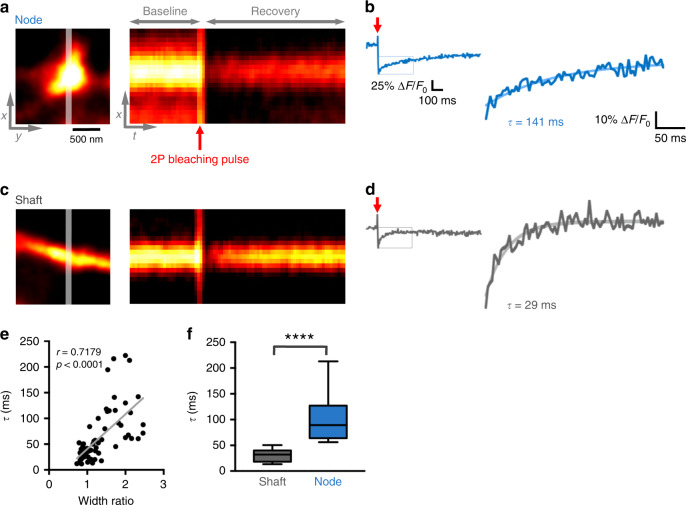
Astrocytic nodes are biochemically compartmentalized. **a**, **c** An example of node (**a**) and shaft (**c**). White line indicates the site of imaging and 2P bleaching (left). Fluorescence recovery over time. Red arrow indicates the timing of 2P bleaching (right). **b**, **d** FRAP traces obtained from experiments shown in (**a**, **c**), showing the recovery of fluorescence normalized to the fluorescence before bleaching. Single exponential equation used for the fit is overlaid with the original trace. **e** Correlation between width ratio (Node or shaft width at the bleach location/shaft width 1 μm away from the bleach location) and *τ* (*n* = 66 structures from 7 slices; Spearman *r* = 0.7179, ****p (two-tailed) < 0.0001). Higher width ratio was associated with longer *τ*. **f** Comparison of *τ* between nodes (*n* = 27) and shafts (*n* = 39), indicating that the anatomical parameters of nodes shaped the degree of compartmentalization (Mann–Whitney *U* test, ****p (two-tailed) < 0.0001,). Data are presented as median, interquartile range, and whiskers 10–90%.

Given their bulbous shape, we wondered whether nodes could function as diffusionally isolated compartments, similar to mushroom-like spines or en passant axonal boutons, which are known to compartmentalize biochemical signals. We tested this by performing fluorescence recovery after photobleaching experiments (FRAP) in nodes and shafts (Fig. 5a–d).

We applied brief two-photon laser pulses to bleach an appreciable fraction (~40%) of the ZsGreen fluorescence inside astrocytic processes and recorded the fluorescence recovery by confocal imaging in rapid line-scan mode. The time constant of recovery (*τ*) quantifies the time it takes for the cytosolic fluorescent marker to equilibrate after a local drop in concentration. *τ* is thus a measure of biochemical compartmentalization, which should scale with the volume of the node and the diffusional resistance of the shafts^27^. We estimated *τ* by fitting the fluorescence traces with a mono-exponential decay function and calculated the width ratio (width at bleaching site divided by width of shaft), which becomes unity in the absence of a node.

Plotting *τ* against the width ratio (Fig. 5e), we found a strong positive correlation, where nodes with high width ratios (>2) had on average at least a twofold higher *τ* than structures with smaller ratios (<1.5). This indicates that micro-anatomical features give nodes the ability to compartmentalize biochemical signals (Fig. 5f).

### Nodes exhibit spatially confined Ca^2+^ transients

**Fig. 6:**
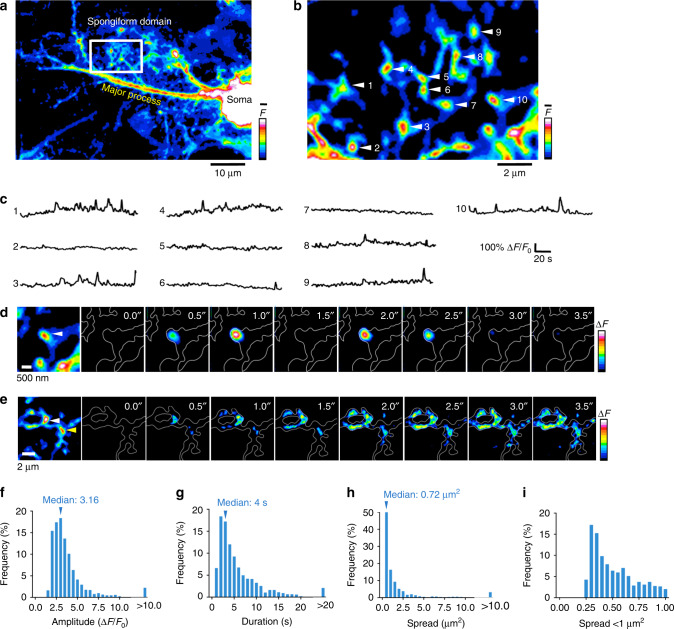
Astrocytic nodes exhibit microdomain Ca^2+^ signals. **a** A confocal overview image of a GCaMP6s-expressing astrocyte. **b** A zoom image of a box in (**a**). ROIs were manually placed on typical nodes. The general anatomical layout of shafts and nodes are still recognizable in confocal images. Nodes typically appear as bright spots at branch points. **c** Spontaneous Ca^2+^ traces from ROIs shown in **b**, showing that each node exhibits unique pattern of Ca^2+^ signals. **d** Time-lapse images of Ca^2+^ events, which were spatially restricted to a single node (right), and the corresponding confocal image of the structure where node is indicated with a white triangle (left). **e** Time-lapse images of a Ca^2+^ event, which originated at a node and spread via the connecting shafts to a neighboring node (right), and the corresponding confocal image of the structure where the originating node is indicated with a white triangle and neighbor node is indicated with a yellow triangle (left). **f** Frequency distribution of amplitude of Ca^2+^ events (*n* = 1583 events from 5 slices). **g** Frequency distribution of duration of Ca^2+^ events (*n* = 1583 events from 5 slices). **h** Frequency distribution of spread of Ca^2+^ events. In 59% of the events, the spread was less than 1 µm^2^ (*n* = 1583 events from 5 slices). **i** Frequency distribution of spread of Ca^2+^ events under 1 µm^2^, suggesting that the majority of Ca^2+^ events in the spongiform domain were confined to local domains corresponding to single nodes (*n* = 941 events from 5 slices).

Having shown that nodes are distinct physical compartments in contact with spines, we studied their capacity for Ca^2+^ signaling. Expressing the Ca^2+^ sensor GCaMP6s in astrocytes, we performed confocal Ca^2+^ imaging at 2 Hz. With the STED laser switched off, the spatial resolution of our microscope was diffraction-limited to around 200 nm in x–y and around 600 nm in z. Nevertheless, it was still possible to recognize the anatomical layout of shafts and nodes (Fig. 6a), the latter appearing as bright spots at branch points in the resting fluorescence of GCaMP6s (Fig. 6b).

We could detect spontaneous Ca^2+^ activity with a high signal-to-noise ratio in the nodes (Fig. 6b–e), indicating their Ca^2+^ signaling competence. While most Ca^2+^ signals appeared to be restricted to single node-like structures (Fig. 6b–d), they sometimes also spread to neighboring ones (Fig. 6e).

Analyzing the spatial spread and time course of all Ca^2+^ transients (detectable by Ca^2+^ imaging at 2 Hz) in the spongiform domain with an automated Ca^2+^ detection software, we measured a median amplitude (∆*F*/*F*_0_) of 316% (Fig. 6f) and a median duration of 4 s (Fig. 6g). The median area was 0.72 µm^2^ and 59% of the events were smaller than 1 µm^2^, indicating that the majority of spontaneous astrocytic Ca^2+^ events was confined to individual microdomains (Fig. 6h, i).

By contrast, in the major astrocytic branches (with diameters > 1 μm), Ca^2+^ transients were larger both in amplitude and spatial spread (median ∆*F*/*F*_0_ = 433%; median area = 2.15 µm^2^) (Supplementary Fig. 4a–e), indicating consistent differences in Ca^2+^ signals between the spongiform domain and larger astrocytic compartments.

**Fig. 7:**
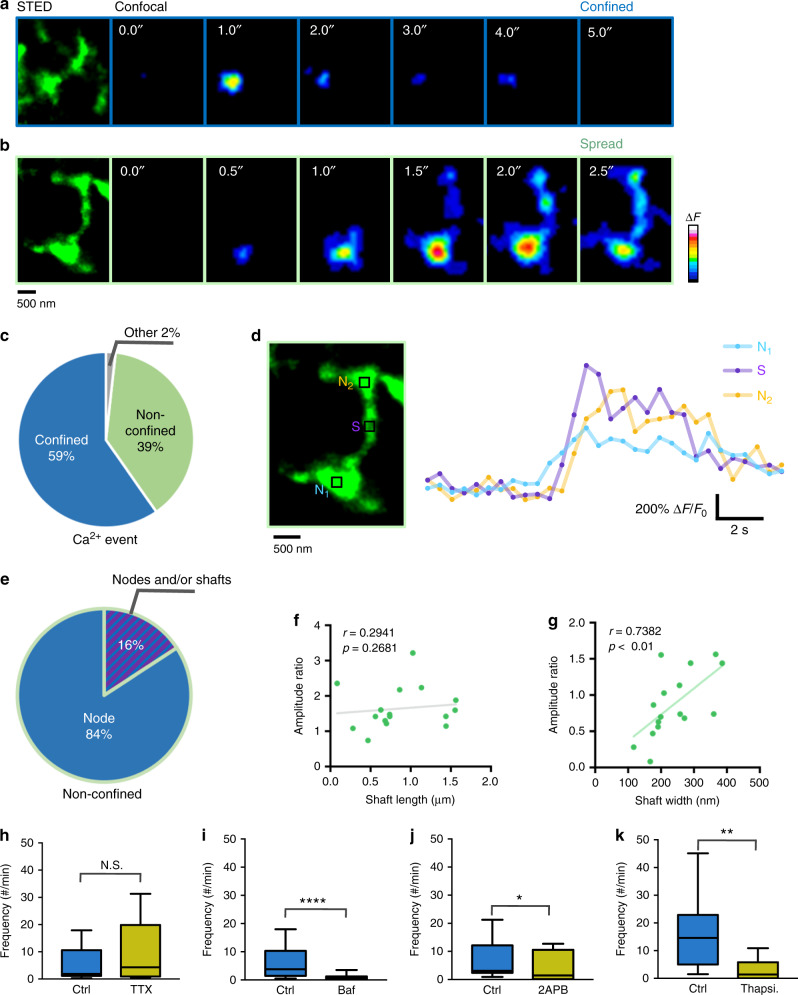
Nodes are sites of initiation of Ca^2+^ signals in astrocytic processes. **a** Confocal time-lapse images of a spontaneous Ca^2+^ event that was confined to a single node (right) and the corresponding STED image of the underlying structure (left). **b** Confocal time-lapse images of a non-confined spontaneous Ca^2+^ event (right) and the corresponding STED image (left). **c** Percentage of Ca^2+^ events that were confined to single nodes, non-confined and that occurred at other undefined structures. The majority of spontaneous Ca^2+^ events are confined (*n* = 516 events from 11 slices). **d** Ca^2+^ traces of the non-confined Ca^2+^ event described in **b** (right) from ROIs indicated on the corresponding STED image (left). N_1_: node that initiated the Ca^2+^ event, S: neighbor shaft, N_2_: connected neighbor node. **e** Percentage of Ca^2+^ events that were initiated at nodes, and those where the point of initiation could not be determined node and/or shaft, among the non-confined Ca^2+^ events (*n* = 19 events from 9 slices). **f** Correlation between Amplitude ratio (amplitude at neighbor node/amplitude at initiation node) and interconnecting shaft length. The spread of the Ca^2+^ signal did not depend on shaft length (*n* = 16 events from 7 slices; Spearman *r* = 0.2941, *p* (two-tailed) = 0.2681). **g** Correlation between amplitude ratio and interconnecting shaft width. The spread of the Ca^2+^ signal was correlated with shaft width (*n* = 16 events from 7 slices; Spearman *r* = 0.7382, **p (two-tailed) = 0.0016). **h** Frequency of Ca^2+^ events under control and TTX (1 µM) conditions. (*n* = 14 cells from 4 slices for both conditions; Mann–Whitney *U* test *p* (two-tailed) = 0.38, N.S., not significant). Data are presented as median, interquartile range, and whiskers 10–90%. **i** Frequency of Ca^2+^ events under control and Bafilomycin A1 (Baf, 2 µM) conditions. The frequency was significantly reduced by bafilomycin A1, suggesting that miniature synaptic events triggered the Ca^2+^ transients (*n* = 21 cells from 4 slices for control and *n* = 23 cells from 4 slices for Bafilomycin A1 condition; Mann–Whitney *U* test, ****p (two-tailed) < 0.0001). Data are presented as median, interquartile range, and whiskers 10–90%. **j** Frequency of Ca^2+^ events under control and 2APB (100 µM) conditions. 2APB significantly decreased frequency of Ca^2+^ transients, indicating the involvement of IP_3_Rs (*n* = 12 cells from 4 slices for both conditions; Mann–Whitney U test, *p (two-tailed) = 0.0425). Data are presented as median, interquartile range, and whiskers 10–90%. **k** Frequency of Ca^2+^ events under control and Thapsigargin (Thapsi, 2 µM) conditions. Thapsigargin significantly decreased frequency of Ca^2+^ transients, indicating the involvement of internal Ca^2+^ stores (*n* = 13 cells from 3 slices for control and *n* = 15 cells from 3 slices for Thapsigargin condition; Mann–Whitney *U* test, **p (two-tailed) = 0.0017). Data are presented as median, interquartile range, and whiskers 10–90%.

To directly link Ca^2+^ signals and astrocytic structures, we combined confocal Ca^2+^ imaging with STED microscopy. STED measurements of the resting GCaMP6s signal confirmed that the bright spots indeed were nodes (Supplementary Fig. 5a–c, Fig. 7). By rapidly changing the imaging modality, we could map the Ca^2+^ signals onto the nodes and shafts.

Analyzing the spatio-temporal pattern of the Ca^2+^ activity, we found that the majority of Ca^2+^ events (59%) was confined to single nodes, while the rest also involved neighboring shafts and nodes (Fig. 7a–c) in addition to 2% where the structure was unidentifiable. Notably, we never detected any events that were restricted to shafts only.

To examine the temporal order of the Ca^2+^ events that involved multiple neighboring nodes, we determined the onset time of the Ca^2+^ transients in neighboring shafts and nodes (Fig. 7d). For the vast majority of cases (84%), the Ca^2+^ signal first appeared at a given node, before becoming detectable in the neighboring structures, while for the remainder (16%), the temporal order could not be resolved (Fig. 7e). While the temporal pattern is suggestive of a propagating wave of intra-astrocytic Ca^2+^, it could also reflect a sequential activation of the nodes by some external signal.

Next, we examined the Ca^2+^ events in neighboring nodes where there was a clear temporal order. The amplitude ratio of the events depended on the width, but not the length, of the connecting shaft (Fig. 7f, g), supporting the idea of a propagating wave whose passage from node to node depends on the nanoscale design of the astrocytic processes.

These observations were confirmed by (four-fold) faster Ca^2+^ imaging (8 Hz) (Supplementary Fig. 5d), revealing a clear sequential latency for the onset time of the Ca^2+^ transients in neighboring shafts and nodes (Supplementary Fig. 5d, f). The majority (66%) of all detectable Ca^2+^ events were confined to a single node (Supplementary Fig. 5e), while 83% of the remaining non-confined events had initiated at a single node (Supplementary Fig. 5g). Not surprisingly, the median duration of the events was much shorter for 8 Hz than for 2 Hz Ca^2+^ imaging (Supplementary Fig. 5h), presumably because more short-lived events were detected by the higher acquisition speed, consistent with a recent report^15^.

Having identified the nodes as a primary site of Ca^2+^ activity in the spongiform domain, we investigated the molecular mechanism underlying the Ca^2+^ transients. First, to find out if they depended on neuronal activity, we incubated the slices with TTX (1 µM), which blocks voltage-gated sodium channels and thus action potential firing in neurons, or bafilomycin A1 (2 µM), which disrupts neurotransmitter release by inhibiting vacuolar ATPases on presynaptic vesicles containing neurotransmitter.

While the frequency of Ca^2+^ transients was unaffected by TTX (Fig. 7h), it was significantly reduced in the presence of bafilomycin A1 (Fig. 7i), in line with the previous reports^4,28^. These results suggest that miniature synaptic events triggered the Ca^2+^ transients. However, we cannot rule out drug effects on the potential acidic Ca^2+^ stores^29^ in astrocytes.

To identify the source of astrocytic Ca^2+^ signals, we used 2APB (100 µM), which blocks IP_3_ receptors (IP_3_R) that are the main intracellular Ca^2+^ release channels in astrocytes. 2APB significantly decreased the frequency of Ca^2+^ transients (Fig. 7j), indicating that IP_3_Rs are involved. Furthermore, incubation with Thapsigargin (2 µM), which depletes internal Ca^2+^ stores by inhibiting SERCA, greatly reduced the frequency of Ca^2+^ transients (Fig. 7k).

We also stimulated astrocytes by bath-applying the mGluR agonist DHPG (10 µM) in the presence of TTX (1 µM). DHPG evoked Ca^2+^ signals that initiated at nodes, which provides additional evidence for functional IP_3_Rs there (Supplementary Fig. 6).

### Nodes host Ca^2+^ transients at tripartite synapses

**Fig. 8:**
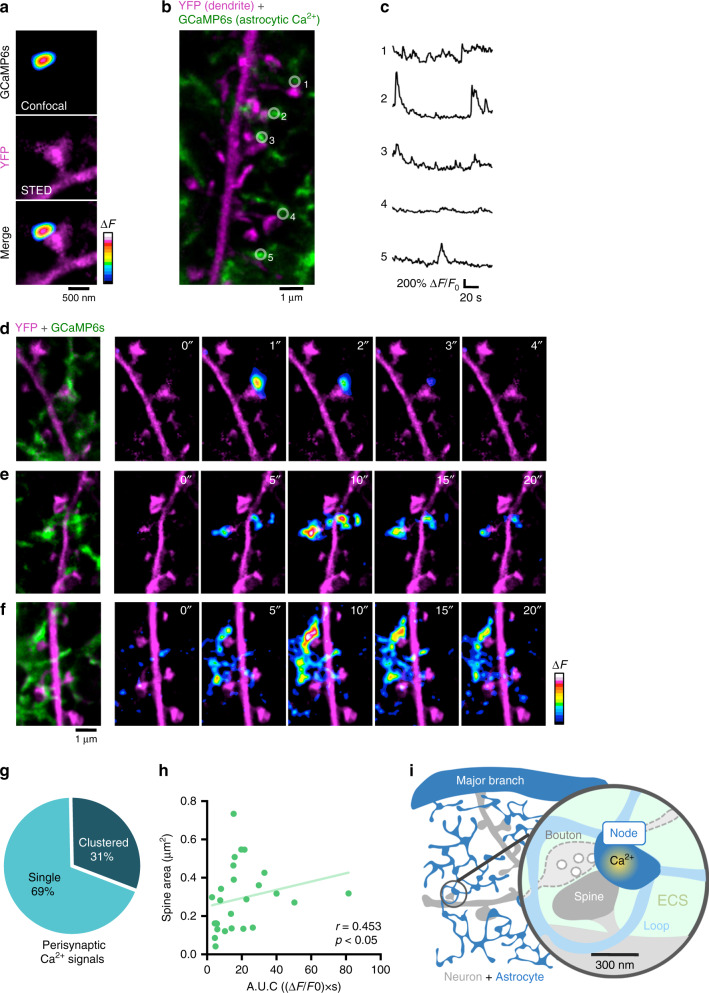
Astrocytic nodes are likely functional component of excitatory tripartite synapses. **a** Confocal Ca^2+^ signal in node mapped onto STED image of spine morphology. **b** A STED image of YFP-labeled neuron and GCaMP6s-expressing astrocyte. **c** Spontaneous Ca^2+^ traces from ROIs shown in **b**. Note that nodes exhibit unique activation patterns. **d**–**f** Left: A STED images of YFP-labeled neurons and GCaMP6s-expressing astrocytes. Right: Ca^2+^ activity mapped onto dendritic morphology, where the Ca^2+^ event remained confined to a single node (**d**), where two perisynaptic nodes were co-active (**e**) or where a Ca^2+^ wave appeared to propagate within the local astrocytic network (**f**). **g** Percentage of perisynaptic Ca^2+^ events confined to single nodes or involving multiple nodes, showing that majority of Ca^2+^ events was confined (*n* = 61 events from 30 slices). **h** Correlation between spine size and area under curve (A.U.C.) of the node Ca^2+^ event. Large spines were associated with large Ca^2+^ events in nodes (*n* = 26 structures from 24 slices; Spearman *r* = 0.453, *p (two-tailed) = 0.0201). **i** Summary schematic of the tripartite synapse micro-environment consisting of a presynaptic bouton, dendritic spine, astrocytic node, shaft, loop and extracellular space (ECS). Nodes appear as the functional astrocytic component of excitatory tripartite synapses, which may provide the anatomical basis for synapse-specific communication.

The finding that spines predominantly formed contacts with nodes and that nodes exhibited Ca^2+^ signals suggests that nodes are the astrocytic element of the tripartite synapse. To demonstrate this more directly, we combined confocal Ca^2+^ imaging in astrocytes with STED microscopy of dendritic morphology. We used slices from Thy1-YFP animals where a subset of neurons express YFP and infected the astrocytes with GCaMP6s. We could map the confocal Ca^2+^ signals onto the morphology of nodes and spines (Fig. 8a–c, Supplementary Movies 2–4), making it possible to examine their spatial relationships and link node Ca^2+^ signals to individual synapses.

Focusing on nodes that were in contact with labeled spines, spontaneous Ca^2+^ events could again be readily detected. The majority of the Ca^2+^ signals were confined to single nodes (Fig. 8d, g), but sometimes also occurred synchronously or propagated across multiple nodes. In the latter cases, the Ca^2+^ activity appeared to associate with clusters of nearby spines on the same dendrite (Fig. 8e–g), suggesting that astrocyte-synapse crosstalk can also occur on a wider scale, involving dendritic segments in an orchestrated way.

Moreover, the area under the curve (A.U.C.), which is a measure of the cumulative Ca^2+^ activity in the nodes, was positively correlated with spine size, lending additional support to the idea that nodes are functionally coupled to their partner spines (Fig. 8h).

Taken together, these results suggest that nodes are a primary site for Ca^2+^ signaling at excitatory tripartite synapses, providing the structural framework and molecular machinery for synapse-specific communication between astrocytes and neurons (Fig. 8i).

## Discussion

Our study brings to light several new and striking aspects of the micro-anatomical organization and Ca^2+^ activity of astrocytes, sharpening our understanding of the tripartite synapse, which is a major concept in the neuron-glia field^3^.

The STED images reveal a spongiform domain, which is characterized by a dense network of nodes and shafts that come into close but not consummate physical contact with synaptic structures. Our data indicate that the morphological association may be much less tight in a live setting than what has been inferred from EM images of chemically fixed tissue, where astrocytic structures appear to engulf synapses^2,30,31^.

Previous studies reported that astrocytic processes can be relatively motile near synapses^32,33^, whereas our data show that node-spine contacts are stably maintained at the nanoscale for at least a couple of hours under baseline conditions. This discrepancy may be due to methodological differences (spatial resolution, sample stability), or reflect the fact that we focused on nodes that were in close contact (<100 nm) with spines, and not on potentially unmoored astrocytic processes. Conceivably, the contact stability we observed is provided by puncta adhaerentia that mechanically connect dendritic spines to neighboring astrocytic processes^34^.

Our 3D-STED approach marks a major step forward in the visualization of astrocytes in living brain tissue, but it may still do injustice to some aspects of astrocytic morphology, such as sheet-like structures, which have been reported by EM^35^. Cytosolic labels in hyper-thin sheets or plasmalemmal films can generate only a weak fluorescence signal, while membrane labels tend to quickly suffer from photobleaching because they do not recover by replenishment, posing a challenge for live-cell super-resolution microscopy.

That said, a recent EM study based on high-pressure freezing of tissue slices^22^ also showed a relatively loose organization of rotund astrocytic structures close to synapses, suggesting that classic chemical fixation via cardiac perfusion introduces artifacts by compacting the tissue. In agreement with the cryogenic EM study, the nodes and shafts we observed had simple geometries, reminiscent of axonal boutons and fibers, and sizes that were well within the STED resolution. Moreover, the SUSHI images indicate that the extracellular space is relatively spacious around synapses, in line with biophysical measurements, which have reported extracellular space volume fractions of around 20% in living brain tissue^36^.

It is important to know these structural details, because the relative position, size, and shape of astrocytic processes will influence their ability to communicate with their synaptic partners^37^.

The nodes and shafts are morphologically recognizable in several brain areas and experimental preparations. However, additional improvements in microscopic resolution and sensitivity may still be needed to provide a definitive view of astrocytic morphology in vivo. They will facilitate mechanistic analyses of the structural and functional dynamics of neuron-astrocytes interactions and along the way help settle controversies about the effects of EM protocols on tissue ultrastructure.

Astrocytes are thought to regulate synapses via Ca^2+^ signaling in their perisynaptic processes. However, it has remained technically challenging to record Ca^2+^ transients and to map them onto the underlying cellular structures^18^. We have demonstrated here that a combination of confocal Ca^2+^ imaging and STED microscopy can achieve this.

We found that a large fraction of spontaneous Ca^2+^ signals in the spongiform domain were confined to single nodes and rarely spread into the intervening shafts, even when neighboring nodes were co-active. Given the dense spacing of the nodes (~1 micron), this indicates an extremely tight spatial compartmentalization, and speaks to the need for advanced imaging techniques that can resolve on a sub-micron scale the Ca^2+^ signals and the structures that host them^18^.

We cannot rule out that shafts are also capable of generating Ca^2+^ events, given the limited sensitivity and temporal resolution of our experimental approach, which may have failed to detect very small and/or brief Ca^2+^ events. Yet, as it stands, our data indicate that Ca^2+^ events are primarily associated with nodes. For the same technical reason, we may have missed near-membrane Ca^2+^ events in astrocytic sheets.

Our study revealed that spongiform Ca^2+^ events in part depended on IP_3_Rs, which is consistent with the previous studies^4,12^, but Ca^2+^ influx from the extracellular space as well as mitochondria may also have contributed to the Ca^2+^ events^16,38^. Given their size, which is comparable to dendritic spines (Supplementary Fig. 3a), nodes should be able to accommodate the molecular signaling machinery composed of IP_3_Rs, Ca^2+^ channels, and mitochondria, which generates Ca^2+^ signals and maintains Ca^2+^ homeostasis^39^.

Our FRAP and Ca^2+^ imaging data indicate that the anatomical design of the astrocytes is an influential determinant of the spread of signaling molecules or ions like Ca^2+^, raising the possibility that astrocytes might use structural changes to dynamically regulate intracellular signaling.

Given the association between nodes and spines, our Ca^2+^ data support the idea that astrocytes can have many private conversations in parallel with different synapses, i.e. without much mutual interference between the tens of thousands of synapses a single astrocyte is presumably in contact with. Together with recent work showing rapid Ca^2+^ signals in astrocytes^4,5,9,15^, our study makes an important step towards the investigation of the activity-dependent and synapse-specific regulation of brain circuits by astrocytes, which is an important—albeit unproven—implication of the tripartite synapse.

In the spongiform domain, astrocytes are characterized by a complex meshwork of interconnecting shafts and nodes that shows little structural polarity or gradient, unlike dendritic and axonal trees of neurons, whose branches are hierarchically organized and tend to taper distally. Strikingly, in the STED images, we observed circular structures. For cell morphological structures to form genuine rings, their membranes must fuse or split, as budding or squishing alone will not do it. For example, blood vessels grow capillary beds by intussusception where blood vessels form holes before splitting up^40^, and cellular organelles like endoplasmic reticulum can fuse with each other^41^. However, neuronal cell membranes apparently do not have that ability, as daughter branches of dendrites or axons never reconnect.

This raises interesting cell-biological questions about which structures can fuse and how they do it. Equally intriguing is the functional significance of the rings; they might mechanically constrict the structures that are inside or link up astrocytic processes to form anastomotic-like networks like capillary beds of the vasculature. Moreover, if astrocytic processes can fuse with each other, they might create shortcuts or roundabouts, which could affect the way signaling molecules can spread within the spongiform domain of astrocytes.

In conclusion, our super-resolution approach revealed a unique anatomical organization of astrocytic processes, featuring nanoscale subcellular signaling domains tailored for synapse-specific communication between neurons and astrocytes.

## Methods

### Experimental animals

All experiments were performed using mouse organotypic hippocampal slice cultures, with the exception of the data presented in Fig. 2 that were obtained from acute slices and the brain in vivo. Experimental procedures were in accordance with the French National Code of Ethics on Animal Experimentation and approved by the Committee of Ethics of Bordeaux. All procedures were in accordance with the guidelines of the European Directive 2010/63/UE.

Mice were housed under a 12 h light/12 h dark cycle at 20–22 °C with ad libitum access to food and water in the animal facility of the Neurocentre Magendie (Inserm/the University of Bordeaux), and monitored daily by trained staff. All animals used were free of any disease or infection at the time of experiments. Pregnant females and females with litters were kept in cages with one male. We did not distinguish between males and females among the perinatal pups used for organotypic cultures, as potential anatomical and/or physiological differences between the two sexes were considered irrelevant in the context of our study. For in vivo experiments, only male mice were used. No animals used had been involved in previous experiments.

### Mouse lines

Slices with ZsGreen-labeled astrocytes were obtained by cross-breeding tamoxifen inducible heterozygous GFAP-cre (Tg(GFAP-cre/ERT2)1Fki^42^) and homozygous Ai6 (RRID: IMSR_JAX:007906) mice. For slices with YFP-labeled neurons, pups were produced by cross-breeding C57Bl/6J (RRID: IMSR_JAX:000664) with homozygous Thy1-YFP-H (RRID: IMSR_JAX:003782) mice. We used C57Bl/6 J wild-type for imaging in organotypic and acute slices as well as in vivo.

### Cloning and virus production

To generate Sindbis-Citrine, the Citrine sequence (Addgene Plasmid #54715) was inserted to the pSinRep5 plasmid (Invitrogen, K750-01). AAV2/1.gfaABC_1_D-Clover and AAV2/1.gfaABC_1_D-Lck-Clover were generated by replacing *GFP* gene of pZac2.1-gfaABC_1_D-GFP, and pZac2.1-gfaABC_1_D-*Lck-GFP* with the *Clover* sequence (Addgene plasmid #40259).

### Viral infection of organotypic slices

Microinjection of virus into the hippocampal slice was performed using a glass pipette connected to Picospritzer (Parker Hannifin)^43^. In brief, the virus was injected via a pipette positioned into the CA1 area of the slice by brief pressure pulses (40 ms; 15 psi). For morphological or Ca^2+^ imaging of astrocytes, AAV2/1.gfaABC_1_D-Lck-Clover, AAV5-gfaABC1D-Lck-GCaMP6f (gift from Baljit Khakh; Addgene viral prep # 52924-AAV5) or AAV9-GFAP-GCaMP6s^9^ was microinjected into the stratum radiatum of 2-week old slices from either WT mice or Thy1-YFP-H (JAX:003782) mice 4 to 6 weeks before the experiment. For labeling of neurons in the organotypic preparation, Sindbis-Citrine virus were microinjected into slices with ZsGreen-labeled astrocytes 1 day prior to the experiment.

### In vivo injections

Viral delivery into the hippocampus was performed by in vivo injection^6^. In brief, 4-week old mice were submitted to stereotaxic surgery and AAV2/1.gfaABC_1_D-Clover was injected into the hippocampus or barrel cortex with the help of a microsyringe attached to a pump. Animals were used 4 to 5 weeks after injections for acute slice and in vivo experiments.

### Extracellular labeling for SUSHI

Extracellular labeling was performed as follows^23^. Organotypic slices were transferred on their glass coverslip to a heated and perfused imaging chamber. The extracellular dye, 40 µM Atto-514 (Atto Tec) diluted in ACSF, was pipetted directly into the chamber while pausing the perfusion. The final fluorophore concentration was around 20 µM.

### Organotypic slices

Organotypic slices were isolated from 5 to 7-day old mouse pups and cultured using the roller tube method^44^. Mouse pups were quickly decapitated and hippocampi were dissected out. They were subsequently cut in 350 µm coronal slices using a tissue chopper (McIlwain), and individual slices were placed on poly-L-lysine coated cover slips. To let the slices stick to the cover slips, they were embedded in chicken plasma and thrombin, which coagulated upon mixing. The coverslips with the slices were transferred to flat-bottomed culturing tubes with medium and placed in a roller drum rotating until the experiment. For experiments, a slice on a coverslip was mounted in a perfused imaging chamber, and the slice was imaged from below through the glass coverslip.

### Acute hippocampal slices

Acutely prepared hippocampal slices were obtained from 8 to 9-week old mice. Mice were anesthetized with isoflurane prior to decapitation. Brains were quickly removed and placed in ice-cold, oxygenated sucrose-based ACSF. 350 µm sagittal slices were cut and incubated for 1 h at 33 °C in carbogenated ACSF. Subsequently, slices were stored at room temperature and used within 4 h after preparation. Experiments were performed in a submerged recording chamber at 33 °C with continuous perfusion of carbogenated ACSF^26^.

### Cranial window surgery

Cranial window was made from 8 to 9-week old mice^45^. Mice were intraperitoneally and intramuscularly injected with buprecare and dexamethasone respectively 25 min before anesthesia induction at 4% isoflurane. Mice were maintained under anesthesia at 1.5% isoflurane, and subsequently placed on a heating pad. An anal probe to measure body temperature was inserted and eye ointment was applied. Mice were injected subcutaneously with Lidocaïne 2% and the skin was treated with Lidocaïne 2% cream. The area for incision was disinfected with betadine and the incision was made in the skin. The surface of the skull was cleaned of any remaining tissue and roughened for adhesion of the liquid glue fixing the head holder to the skull. A helmet from dental cement was created for a stable fixation of the head holder and a craniotomy of 3 mm in diameter was performed above the barrel cortex. The opened skull was rinsed with cortex buffer (123 mM NaCl, 5 mM KCl, 10 mM glucose, 10 mM Hepes, 2 mM CaCl_2_, and 2 mM MgSO_4_; at pH = 7.3) and a glass coverslip with a 3 mm diameter was gently positioned on the brain and sealed with gel glue. The glue was left to dry for 10 min, after which mice were placed below the objective of the microscope for acute imaging.

### STED microscopy

In the case of the one-photon system^23^, the excitation light was provided by a pulsed diode laser (λ = 485 nm) and the STED light came from a Ti:Sa femtosecond laser emitting at 838 nm, which was converted to a wavelength of 597 nm by an optical parametric oscillator (OPO). A signal from the OPO was used to trigger pulsed diode laser. We used a glycerol objective with a correction collar to reduce spherical aberrations. In the case of the 2P-STED setup, the excitation light was provided by a second Ti:Sapphire laser tuned to 910 nm running at 80 MHz that was synchronized to the OPO pulses. We used water-immersion objectives with long working distances, either from Nikon (CFI Apo 60X W NIR, 1.0 NA, 2.8 mm WD)^46^ or Olympus (LUMFI 60×, 1.1 NA, 1.5 mm WD)^45^. The STED pulses were broadened in time by passing them through glass rods and a long optical fiber (20 m) to ensure effective de-excitation and maximal resolution enhancement. Synchronization and optimal pulse delay were achieved with phase-locked loop electronics.

The STED beam reflected twice on two different surfaces of a single spatial light modulator (SLM) (Easy3D Module, Abberior Instruments, Göttingen, Germany) to generate doughnut and bottle beams for 2D and 3D-STED microscopy. The SLM was conjugated to the beam scanner (Yanus IV, TILL Photonics, Gräfelfing, Germany) via appropriate relay lenses and the scanner was conjugated to the back focal plane of the objective via the scan and tube lens combination (F_scan_ = 5 cm and F_tube_ = 25 cm, Leica Microsystems). In the case of 2P-STED microscopy^47^, the STED doughnut was created by passing the STED beam through a vortex phase mask, which imposed a helical 2π phase delay on the wave front. Wave plates (λ/2 and λ/4) were used to make the STED light circularly polarized before it entered into the objective.

The excitation and STED beams were precisely co-aligned using a dichroic mirror and a piezo-positioner. Both beams passed through an x–y beam scanner. The fluorescence was de-scanned and focused into a multimode optical fiber connected to an avalanche photodiode operated in photon-counting mode.

For the FRAP experiments, a second Ti:Sa femtosecond laser beam (at λ = 900 nm) was injected into the optical path of the microscope using a 680 nm short-pass dichroic mirror. For two-color imaging, two fluorophores with partially overlapping excitation and emission spectra (e.g., YFP and GCaMP6s) were imaged simultaneously using a single excitation/STED laser beam pair. The emission signal was split by a 514 nm long-pass dichroic mirror, and was sent into separate photodetectors^48,49^.

### Image acquisition

3D-STED microscopy was used for Figs. 1, 2d, Supplementary Figs. 2b, c, and  6c. All other images except for Fig. 2 were acquired with 2D-STED microscopy. Figure 2 was acquired using 2P-STED microscopy. Z-stacks were typically acquired with these parameters: (1) 20 × 20 × 2 µm^3^, pixel size 19.53 nm, z plane 80 nm × 25 planes for 3D confocal STED images, (2) 20 × 20 × 2 µm^3^, pixel size 19.53 nm, z plane 222 nm × 9 planes for 2D-STED images, (3) 10 × 10 × 1 µm^3^, pixel size 19.53 nm, z plane 250 nm × 4 planes for 2 P images and (4) 10 × 10 × 1.8 µm^3^, pixel size pixel size 19.53 nm, z plane 300 nm × 6 planes for 2P-STED images. For time-lapse 2D-STED imaging, z stacks (5 × 5 × 1.5 µm, pixel size 19.53 nm, z plane 250 nm × 6 planes) were acquired every 30 min for 2.5 h.

2 Hz confocal Ca^2+^ imaging (12.5 × 25 µm^2^, pixel size 100 nm) was performed for 2.5 min and corresponding STED images (12.5 × 25 × 1.5 µm^3^, pixel size 19.53 nm, z plane 250 nm × 6 planes) were acquired subsequently. 8 Hz Ca^2+^ imaging (5 × 10 µm^2^, pixel size 78 nm) was performed for 2 min using bidirectional scanning and corresponding STED images (10 × 5 × 1.2 µm^3^, pixel size 19.53 nm, z plane 100 nm × 12 planes) were acquired subsequently. STED images were always acquired after Ca^2+^ imaging so that Ca^2+^ signals were not affected by the STED laser. Image acquisition was controlled by the software Imspector.

Organotypic slice experiments except for SUSHI (described in Extracellular labelling for SUSHI) were performed in ACSF containing 125 mM NaCl, 2.5 mM KCl, 1.3 mM MgCl_2_, 2 mM CaCl_2_, 26 mM NaHCO_3_, 1.25 mM NaH_2_PO_4_, 20 mM D-glucose, 1 mM Trolox; 300 mOsm; pH 7.4. Perfusion rate was 2 ml per min and the temperature 32 °C. Slices were incubated for 20 min for 1 µM TTX (Ascent scientific) and 100 µM 2APB (TOCRIS), 2 h for 2 µM Bafilomycin A1 (Sigma-Aldrich), 20 min for 2 µM Thapsigargin (TOCRIS) prior to imaging. All drugs except for Thapsigargin were present during the recording. 10 µM DHPG (Sigma-Aldrich) was bath-applied after 3 min of baseline recording for the rest of the recording time (7 min). The same concentration of DMSO was added to the control group for drugs that were dissolved in DMSO (2APB, Bafilomycin, and Thapsigargin).

### FRAP experiments

A STED image of the structure (10× 10 × 1.8 µm^3^, pixel size 19.53 nm, z plane 200 nm × 9 planes) was acquired prior to the FRAP experiment. Bleaching was performed by line scanning the two-photon beam for a period of 8 ms across the astrocytic structure (node or shaft) labeled with ZsGreen, concomitant with 488 nm single-photon excitation line scanning to read out fluorescence levels in confocal mode^27^. The bleaching period was preceded by a 0.5 s baseline scan and followed by at least 1 s of scanning to measure recovery. The line scan frequency was 277 Hz, with a pixel size of 23 nm. The time constant *τ* was calculated by fitting a single-exponential function (ImageJ) to fluorescence recovery curve, which was generated by plotting fluorescence intensity versus time^27^. To ensure the good level of fit, only the first 295 ms of the recovery phase was used for the fit. The width ratio was calculated by dividing the measured width at the bleached site by the average width of the two thickest shafts 1 µm away from the bleach site. When the shaft was shorter than 1 µm, the width of the further end of the shaft was used.

### Image processing and analysis

All STED images were deconvolved by Huygens Professional (SVI) software. Loops were manually identified and their inner perimeter and area were measured using ImageJ. Nodes were manually identified based on their enlarged 3D spherical submicron structure and shafts were identified as hyperthin tubes connecting nodes. To measure the width across these astrocytic components, we used a wavelet-based custom-made ImageJ plugin^50^. Distance between nodes was measured as the distance along the shaft from the center of node to the center of the neighboring node. Structures within 20 µm from somatic border were defined as proximal whereas those that were more than 20 µm away were distal.

Two-color images except for Fig. 3d were spectrally unmixed with ImageJ software (NIH) Plugin Spectral unmixing in order to separate spine morphology signals (e.g., YFP, Citrine) and astrocytic morphology/Ca^2+^ signals (e.g., ZsGreen, GCaMP6s). A contact was defined by the presence of at least one pixel overlap between spines and astrocytic components (nodes or shaft). Spine heads were also manually identified and morphological parameters of spines and nodes (perimeter and area) were measured using ImageJ.

Coverage fraction was calculated for all planes that includes a given spine and was subsequently averaged to acquire a representative value. Spines without full 3D information were excluded from the analysis. Coverage fraction for spines was calculated by dividing node-contacting spine head perimeter by total spine head perimeter. Similar measurement was performed to calculate the coverage fraction of boutons. The analysis was performed within the domain of single astrocytes.

The motility index was calculated according to previous studies^33,51^. After cropping out the region of interest, the images were binarized using ImageJ and the average area over time was calculated. Secondly, accumulated area and minimum area were calculated by summating all the time lapse images. Finally, The difference between accumulated area and minimum area was divided by average area to acquire motility index. Fluctuation was calculated as temporal variance of coverage over time.

To analyze Ca^2+^ events in the spongiform structure, Gaussian blur (ImageJ) was applied to the images in order to reduce noise after subtraction of background value, which was calculated by average intensity of the darkest region (typically 5 × 5 pixels) in the field of view. For automatic and unbiased detection and analysis of spontaneous Ca^2+^ events the ImageJ plugin LC_Pro^52^ was used. Detected events were manually verified using Igor Pro (Wavemetrics)^12^. The spatial spread was defined as the maximum best-fit ellipse area above 95% confidence of signal threshold during the event interval. The frequency was calculated by the number of detected Ca^2+^ events in the field of view (12.5 × 25 µm^2^) per minute.

To determine the initiation site of the Ca^2+^ signals in the spongiform domain (Fig. 7, Supplementary Fig. 5d–g), Ca^2+^ events detected by the software were matched with the STED image to identify their location. Ca^2+^ events that were located at nodes were categorized as confined (node) or non-confined (node + shaft). There were no events detected only at the shaft. Ca^2+^ events that occurred on undefined structures was categorized as other. Whether the event was confined or not was determined by the relative size of the Ca^2+^ spread (calculated by LC_Pro) compared with the size of the node that hosted the event. When the Ca^2+^ spread was equal or smaller than the node, it was defined as confined. When Ca^2+^ spread was larger than the node, it was defined as non-confined.

The non-confined group included (i) events that initiated at the node and spread to neighboring structures, and (ii) events where the initiation site could not be determined. To distinguish between these types, we manually placed ROI (2 × 2 pixels) on nodes where LC_Pro had located the event, its neighboring shaft and node (Fig. 7d). Only structures that were included in the Ca^2+^ spread and whose 3D structural information could be reliably captured by STED, were analyzed. We plotted the Ca^2+^ trace using the ImageJ plugin Time Series Analyzer V3. We used the software NeuroMatic^53^ to determine the onset times of the Ca^2+^ transients: after binominal smoothing of the trace, a sigmoid (Boltzmann) function was fitted to the data (achieving a goodness of fit (*R*^2^) of >0.95) to compute the point of maximum curvature, which was defined as the onset time. If the software detected less than 1 frame onset difference between the node and the shaft, it was categorized as Node and/or shaft initiating event. If the onset of node was equal or greater than 1 frame earlier than shaft, then it was categorized as Node initiating event. LC_Pro never detected any events that were initiated on shafts, and we also confirmed this by measuring onset times in the different compartments.

To correlate the shaft morphology with Ca^2+^ signals, only node-initiating events among the non-confined group were analyzed. The amplitude ratio was calculated by dividing the amplitude at N_2_ (neighboring node) by the amplitude at N_1_ (initiating node).

To illustrate the Ca^2+^ changes (Δ*F*) in the figures and movies, we applied a Gaussian filter and the image that preceded the Ca^2+^ event was subtracted from the time series. To map this Ca^2+^ change onto the STED image of dendritic morphology, spine signals in confocal and STED images were used to align these images by ImageJ plugin Align-image-by-line-ROI. Subsequently, the Ca^2+^ change was overlaid with STED spine image. Only Ca^2+^ signals that occurred at perisynaptic nodes, as judged by STED images of astrocytic GCaMP6s and neuronal YFP, were used for the analysis of perisynaptic Ca^2+^ signals. Perisynaptic Ca^2+^ signals were categorized as clustered when other Ca^2+^ events were observed in neighbor perisynaptic nodes along the same stretch of dendrite within the time frame of 2 s. If not, it was categorized as spine-specific.

### Quantification and statistical analysis

Data were tested for normality using D’Agostino–Pearson omnibus test and Shapiro–Wilk test, and comparisons were made by appropriate parametric or non-parametric tests, taking into account number of groups and the nature of the data (paired/non-paired). Statistical tests were performed using Graphpad Prism. The significance level was set at 5%. The size and type of individual samples, n, for given experiments is indicated and specified in the results section and in figure legends. Asterisks indicate p values as follows: *p < 0.05, **p < 0.01, ***p < 0.001.

### Reporting summary

Further information on research design is available in the Nature Research Reporting Summary linked to this article.

## Supplementary information

Supplementary Information

Peer Review

Description of Additional Supplementary Files

Supplementary Movie 1

Supplementary Movie 2

Supplementary Movie 3

Supplementary Movie 4

Reporting Summary

## Data Availability

The data that support the findings of this study are available from the corresponding author upon reasonable request. Further information and requests for resources and reagents should be directed to and will be provided by the lead contact, U.V. Nägerl (valentin.nagerl@u-bordeaux.fr).
