# Author Correction: Structural basis of astrocytic Ca^2+^ signals at tripartite synapses

**DOI:** 10.1038/s41467-020-16453-9

**Published:** 2020-05-18

**Authors:** Misa Arizono, V. V. G. Krishna Inavalli, Aude Panatier, Thomas Pfeiffer, Julie Angibaud, Florian Levet, Mirelle J. T. Ter Veer, Jillian Stobart, Luigi Bellocchio, Katsuhiko Mikoshiba, Giovanni Marsicano, Bruno Weber, Stéphane H. R. Oliet, U. Valentin Nägerl

**Affiliations:** 1 Interdisciplinary Institute for Neuroscience, University of Bordeaux, Bordeaux, France; 2 Interdisciplinary Institute for Neuroscience, CNRS UMR 5297 Bordeaux, France; 3 NeuroCentre Magendie, Inserm U1215, Bordeaux, France; 4 Bordeaux Imaging Center, University of Bordeaux, Bordeaux, France; 5 Bordeaux Imaging Center, CNRS UMS 3420, Bordeaux, France; 6 Bordeaux Imaging Center, INSERM US04, Bordeaux, France; 7 University of Zurich, Institute of Pharmacology & Toxicology, Zürich, Switzerland; 8 ShanghaiTech University, Shanghai, 201210 China

**Keywords:** Super-resolution microscopy, Cellular neuroscience, Astrocyte

Correction to: *Nature Communications* 10.1038/s41467-020-15648-4, published online 20 April 2020

The original version of this Article contained errors in the author affiliations.

Affiliation 1 incorrectly read ‘Interdisciplinary Institute for Neuroscience, University of Bordeaux, Bordeaux, France' instead of the correct ‘University of Bordeaux, Bordeaux, France’.

The affiliation of Misa Arizono with NeuroCentre Magendie, Inserm U1215, Bordeaux France, was inadvertently omitted.

These errors have now been corrected in both the PDF and HTML versions of the Article.

