# Supplementary material for: Structural basis of astrocytic Ca2+ signals at tripartite synapses
Source: Nat Commun. 2020 Apr 20;11:1906. doi: 10.1038/s41467-020-15648-4 (PMC7170846; doi:10.1038/s41467-020-15648-4)
Supplement: Supplementary file 1 — Supplementary Information [file 41467_2020_15648_MOESM1_ESM.pdf]

Supplementary information

Structural basis of astrocytic Ca^{2+} signals at tripartite synapses

Arizono et al.

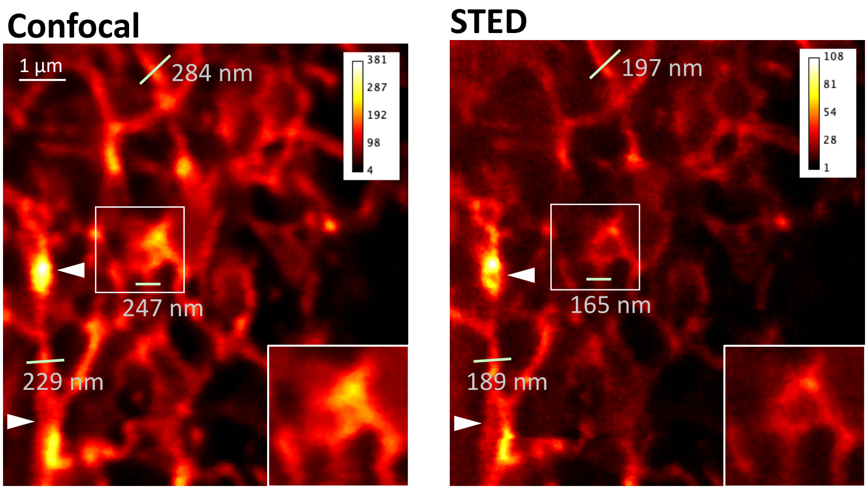

Supplementary Figure 1

Supplementary Figure 1 | Comparison between confocal and STED image
Comparison between confocal (left) and STED (right) image of the identical astrocytic structure.
White triangles and zoom-in images highlight the structural details revealed by the enhanced resolution (n = 3 slices).

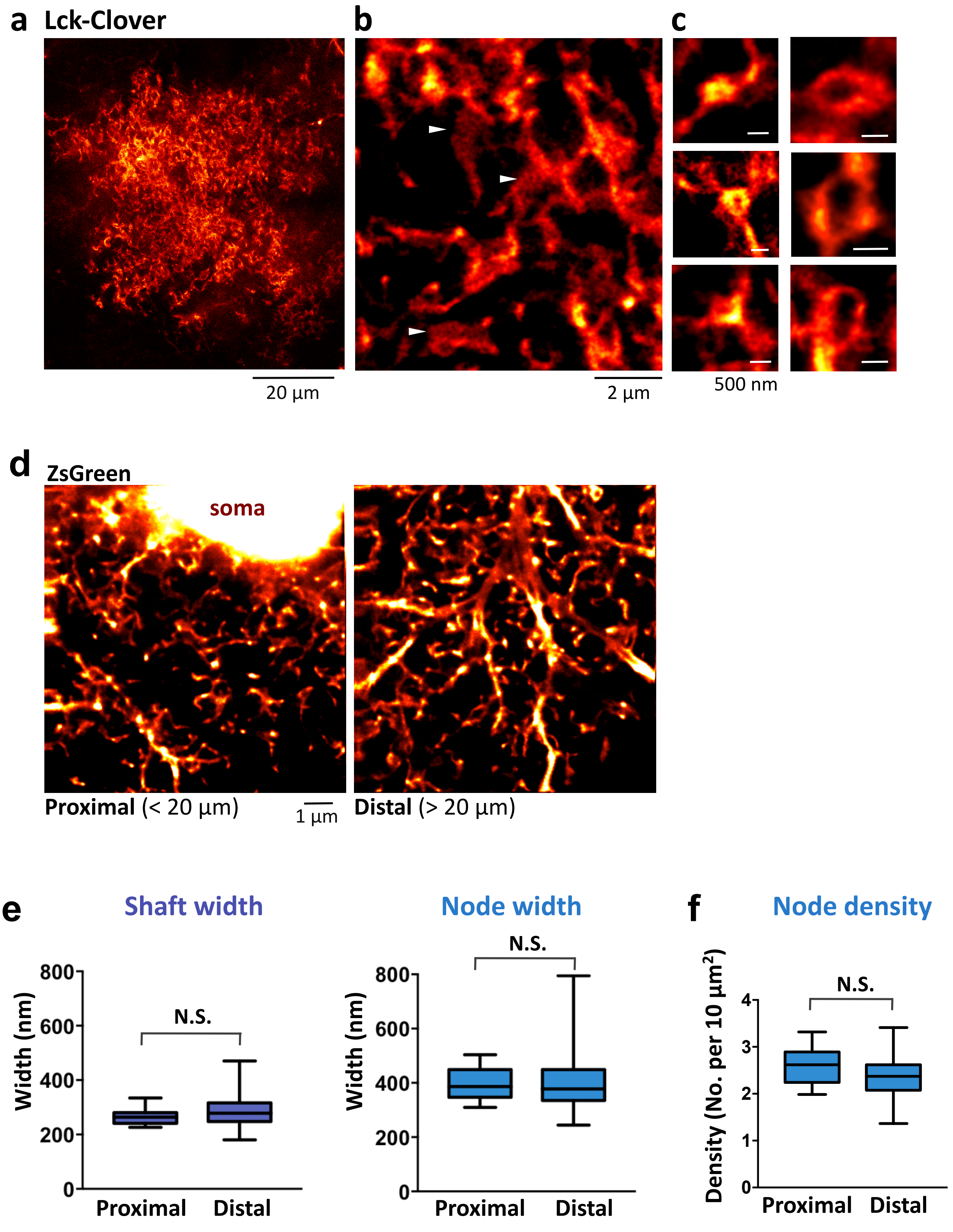

Supplementary Figure 2

Supplementary Figure 2 | STED imaging of astrocytic morphology

(a) Confocal overview image of astrocytes expressing Lck-Clover.

(b) STED images of spongiform domain displaying a reticular meshwork. White arrows indicate sheet-like-structures.

(c) Left: Node-like structures among the spongiform domain. Unlike the cytosolic label, Lck-Clover-labelled nodes appeared less bright and hollow.

Right: Examples of loop-like structures.

(d) Left: A STED image of proximal spongiform domain expressing ZsGreen.

Right: A STED image of distal spongiform domain expressing ZsGreen.

(e) A comparison of width measurements between proximal and distal regions. The anatomical organization of nodes and connecting shafts was equally present in proximal and distal regions (n = 54 for proximal shaft, n = 90 for distal shaft, n = 42 for proximal node, n = 111 for distal node, from 7 slices; Mann-Whitney U test, p (two-tailed) = 0.053 for shafts and p (two-tailed) = 0.70 for nodes, N.S.: not significant). Data are presented as median, interquartile range, and whiskers 10–90%.

(f) A comparison of node density between proximal and distal regions, showing that they are distributed at similar density (n = 13 cells from 7 slices; Mann Whitney U-test p (two-tailed) = 0.30, N.S.: not significant). Data are presented as median, interquartile range, and whiskers 10–90%.

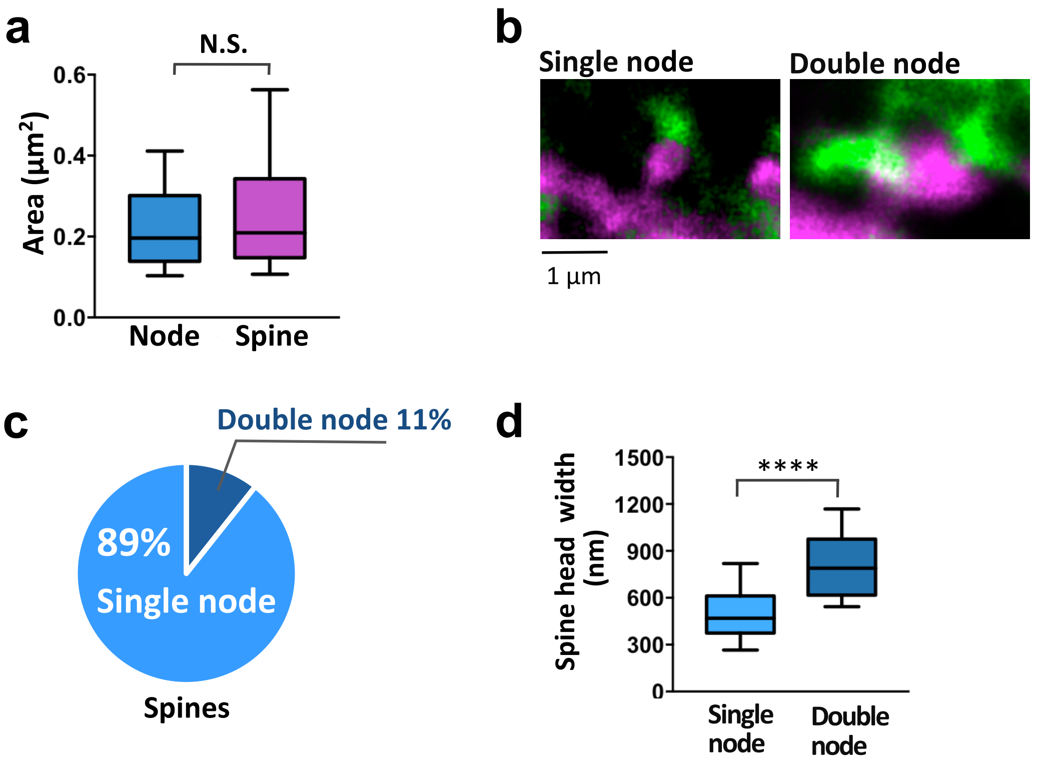

Supplementary Figure 3

Supplementary Figure 3 | Structural relationship between astrocytic nodes and dendritic spines

(a) Comparison of spine head area and astrocytic node area, showing that their sizes are comparable (n = 103 for both components from 21 slices; Mann-Whitney U test, p (two-tailed) = 0.28, N.S.: not significant,). Data are presented as median, interquartile range, and whiskers 10–90%.

(b) Image of spines with one node (left) and two nodes (right).

(c) Percentage of spines with one or two nodes. The majority of spines have one node.

(d) Comparison of spine head width between spines with one and two nodes. Spines with two nodes have wider spine heads (n = 92 for double node spine, n = 11 for single node spine from 22 slices; Mann Whitney U test, **** p (two-tailed) < 0.0001). Data are presented as median, interquartile range, and whiskers 10–90%.

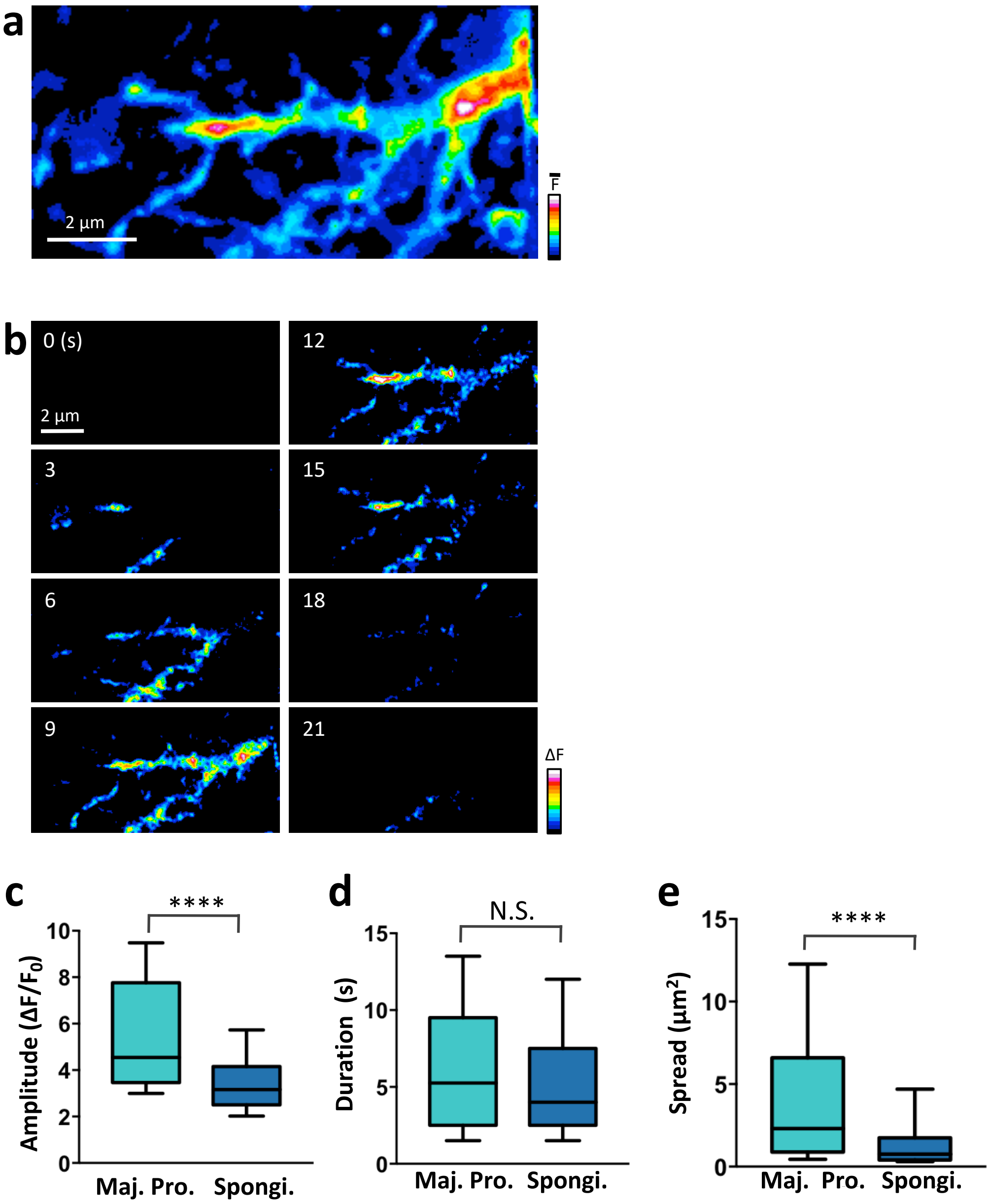

Supplementary Figure 4

Supplementary Figure 4 | Ca^{2+} imaging of astrocytes in major branches

(a) A confocal image showing major branches of GCaMP6s expressing astrocyte.

(b) Time-lapse images of large spontaneous Ca^{2+} waves travelling along major branches.

(c-e) Comparison of amplitude (c), duration (d), and spread (e) of spontaneous Ca^{2+} events between major processes and spongiform domain. In the major astrocytic branches, Ca^{2+} transients had greater amplitudes and were larger in space (n = 60 events for major processes, n = 1583 events for spongiform structure, from 5 slices; Mann-Whitney U test, ****p (two-tailed) < 0.0001, N.S.: Not Significant p (two-tailed) = 0.820).

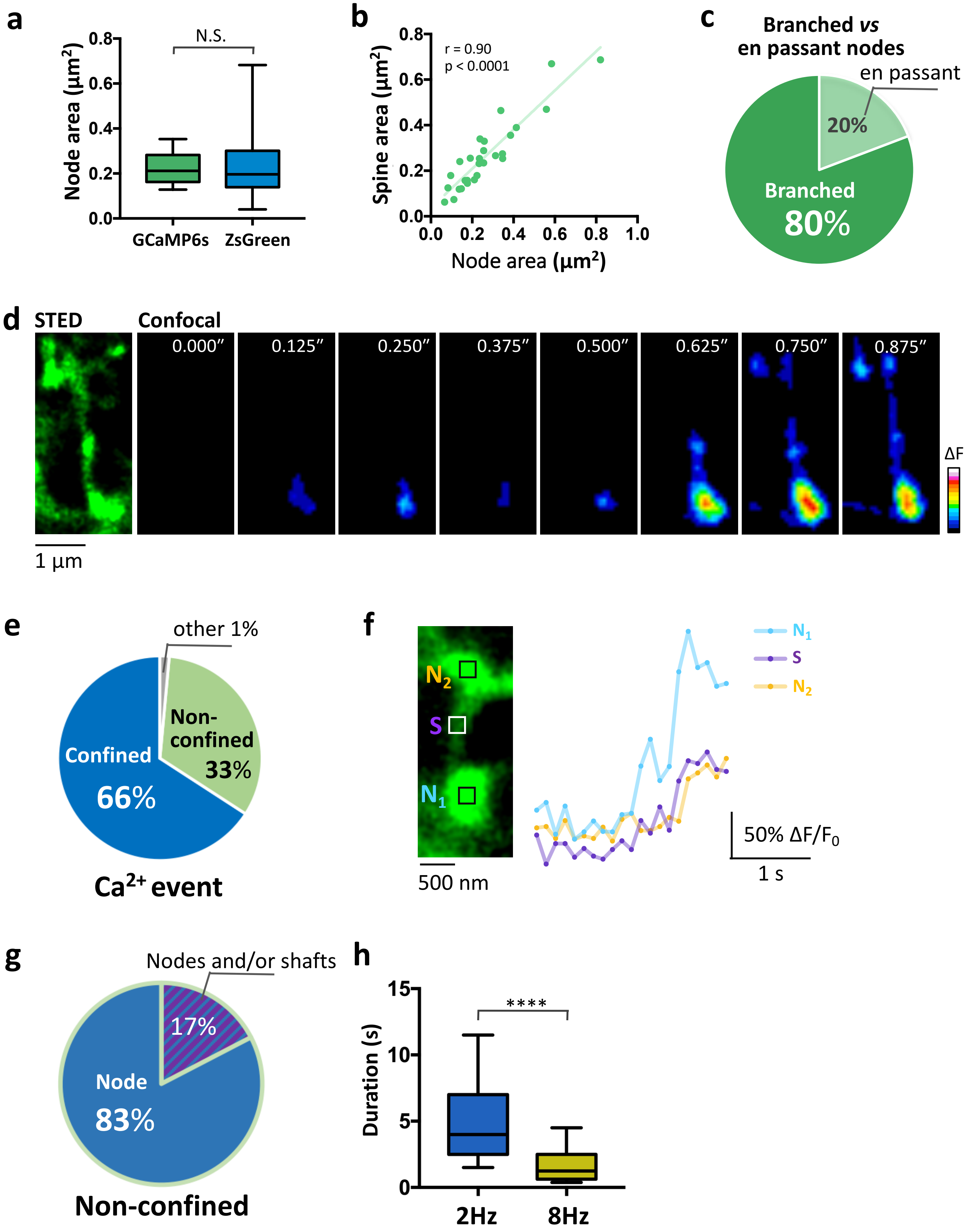

Supplementary Figure 5

Supplementary Figure 5 | Nodes are a prominent site for Ca^{2+} signals in astrocytes

(a) Comparison of GCaMP6s-labelled node size (area) and ZsGreen-labelled node size (n = 52 nodes from 22 slices for GCaMP6s and n = 103 nodes from 21 slices for ZsGreen; Mann Whitney U test, N.S.: Not Significant p (two-tailed) = 0.50). Data are presented as median, interquartile range, and whiskers 10–90%.

(b) Correlation between GCaMP6s-labelled node area and spine head area. Similar to ZsGreen-labelled nodes, GCaMP6s-labelled node area is strongly correlated with spine head area (n = 28 structures from 22 slices; Spearman r = 0.90, **** p (two-tailed) <0.0001).

(c) Percentage of nodes that form branch points. Similar to ZsGreen-labelled nodes, the majority of GCaMP6s-labelled nodes formed branch points (n = 52 nodes from 22 slices).

(d) Time-lapse images of spontaneous Ca^{2+} event acquired at 8 Hz and corresponding STED image of astrocytic process.

(e) Percentage of Ca^{2+} events that were confined to single nodes, non-confined and that occurred at other undefined structures.

(f) Ca^{2+} traces of the Ca^{2+} propagation event (right) from ROIs indicated on the corresponding STED image (left). N_1: node that initiated the Ca^{2+} event, S: neighboring shaft, N_2: connected neighboring node

(g) Percentage of Ca^{2+} events that were initiated at nodes and those that were initiated at nodes and/or shafts, among the non-confined events.

(h) Comparison of Ca^{2+} event duration between Ca^{2+} imaging performed at 2 Hz and 8 Hz (n = 517 events from 11 slices for 2 Hz and n= 627 events from 4 slices for 8 Hz); Mann Whitney U test, **** p (two-tailed) < 0.0001. Data are presented as median, interquartile range, and whiskers 10–90%.

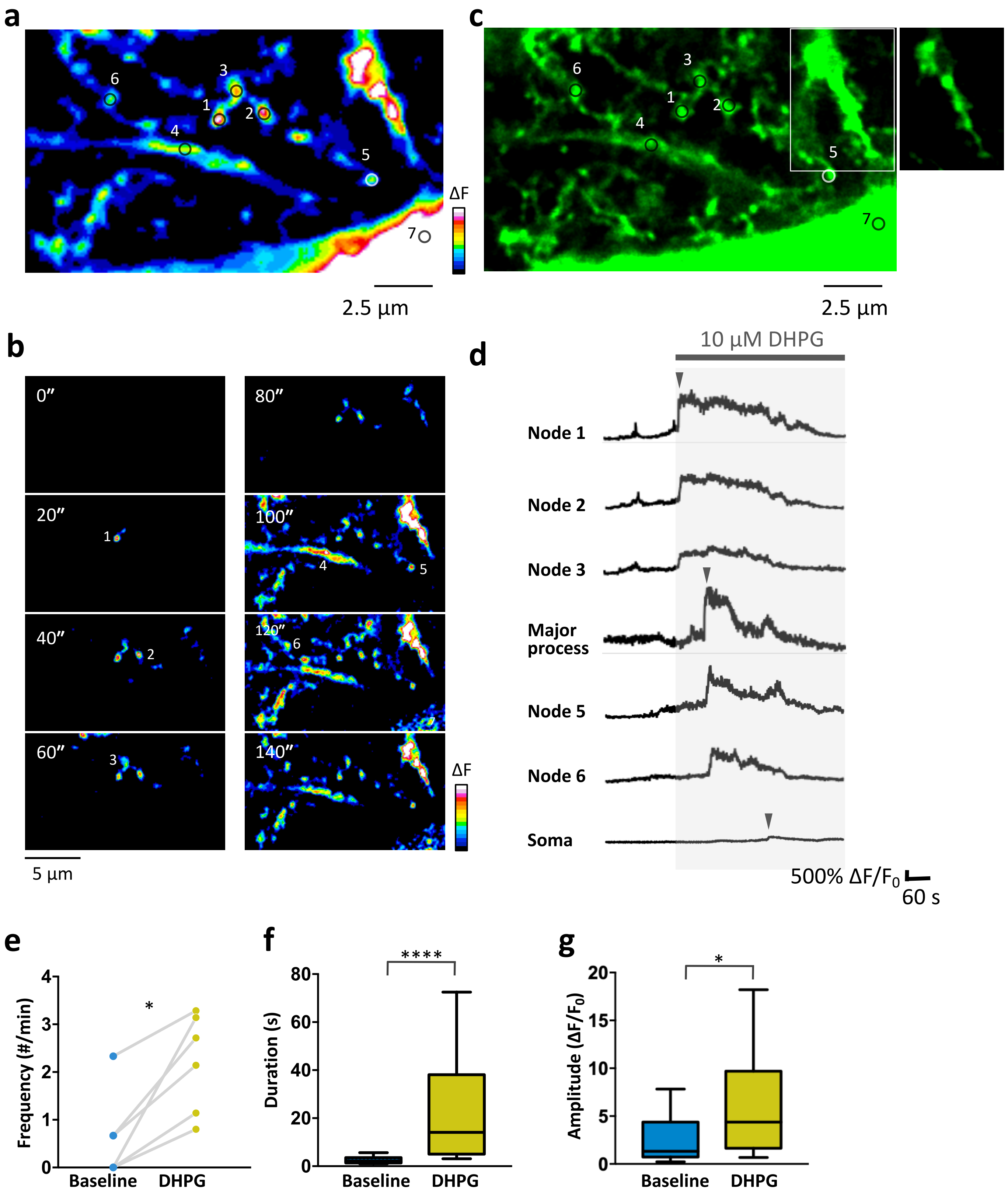

Supplementary Figure 6

Supplementary Figure 6 | mGluR-agonist triggers Ca^{2+} events at the nodes

(a) A confocal overview image of a GCaMP6s expressing astrocyte.

(b) Time-lapse images of a DHPG-evoked Ca^{2+} event, which triggered Ca2+ elevation at node 1, 2 and then 3 leading to wide-spread propagation involving major branch (4) and other nodes (5,6) finally reaching the soma (7). The experiment was performed in ACSF containing 1 μM TTX.

(c) STED image of the underlying structure where the Ca^{2+} signals was recorded from. Note that the structure in the white box appears larger than it really is because of image saturation. Adjusting the brightness level reveals the node and shaft organisation of the astrocytic process.

(d) Ca^{2+} traces from ROIs shown in **b**.

(e) Frequency of Ca^{2+} events per minute during baseline and after DHPG application (n = 6 cells from 6 slices; Wilcoxon matched-pairs signed rank test, * p = 0.031).

(f) Duration of Ca^{2+} events during baseline and after DHPG application (n = 11 events for baseline and n = 89 events for DHPG; Mann Whitney U test, **** p (two-tailed) < 0.0001). Data are presented as median, interquartile range, and whiskers 10–90%.

(g) Amplitude of Ca^{2+} events during baseline and after DHPG application (n = 11 events for baseline and n = 89 events for DHPG; Mann Whitney U test, * p = 0.0174). Data are presented as median, interquartile range, and whiskers 10–90%.
