# Supplementary material for: Structural basis of astrocytic Ca2+ signals at tripartite synapses
Source: Nat Commun. 2020 Apr 20;11:1906. doi: 10.1038/s41467-020-15648-4 (PMC7170846; doi:10.1038/s41467-020-15648-4)
Supplement: Supplementary file 3 — Description of Additional Supplementary Files [file 41467_2020_15648_MOESM3_ESM.docx]

**Description of Additional Supplementary Files**

File name: Supplementary Movie 1
Description: 3D rendering of the ring shown in Fig. 1e

File name: Supplementary Movie 2
Description: Confocal time-lapse images of spontaneous astrocytic Ca2+ signals mapped onto a STED image of dendrite (magenta) and GCaMP6s expressed by astrocytes (grey line)

File name: Supplementary Movie 3
Description: Confocal time-lapse images of spontaneous astrocytic Ca2+ signals and dendrite (magenta) used to generate Supplementary Movie 2

File name: Supplementary Movie 4
Description: Z-stack STED images of dendrite (magenta) and GCaMP6s signal (green) used to generate Supplementary Movie 2
