# Supplementary material for: Structural basis of astrocytic Ca2+ signals at tripartite synapses
Source: Nat Commun. 2020 Apr 20;11:1906. doi: 10.1038/s41467-020-15648-4 (PMC7170846; doi:10.1038/s41467-020-15648-4)
Supplement: Supplementary file 8 — Reporting Summary [file 41467_2020_15648_MOESM8_ESM.pdf]

nature research

Corresponding author(s): UV NÄGERL

Last updated by author(s): 04/03/2020

Reporting Summary

Nature Research wishes to improve the reproducibility of the work that we publish. This form provides structure for consistency and transparency in reporting. For further information on Nature Research policies, see Authors & Referees and the Editorial Policy Checklist .

Statistics

For all statistical analyses, confirm that the following items are present in the figure legend, table legend, main text, or Methods section.

n/a	Confirmed	
☐	☒	The exact sample size (*n*) for each experimental group/condition, given as a discrete number and unit of measurement
☐	☒	A statement on whether measurements were taken from distinct samples or whether the same sample was measured repeatedly
☐	☒	The statistical test(s) used AND whether they are one- or two-sided<br>*Only common tests should be described solely by name; describe more complex techniques in the Methods section.*
☐	☒	A description of all covariates tested
☐	☒	A description of any assumptions or corrections, such as tests of normality and adjustment for multiple comparisons
☐	☒	A full description of the statistical parameters including central tendency (e.g. means) or other basic estimates (e.g. regression coefficient) AND variation (e.g. standard deviation) or associated estimates of uncertainty (e.g. confidence intervals)
☐	☒	For null hypothesis testing, the test statistic (e.g. *F*, *t*, *r*) with confidence intervals, effect sizes, degrees of freedom and *P* value noted<br>*Give P values as exact values whenever suitable.*
☒	☐	For Bayesian analysis, information on the choice of priors and Markov chain Monte Carlo settings
☒	☐	For hierarchical and complex designs, identification of the appropriate level for tests and full reporting of outcomes
☐	☒	Estimates of effect sizes (e.g. Cohen's *d*, Pearson's *r*), indicating how they were calculated

Our web collection on statistics for biologists contains articles on many of the points above.

Software and code

Policy information about availability of computer code

Data collection	Data were collected using Imspector Software (Abberior) (version 0.1).
Data analysis	Deconvolution was performed by Huygens Professional software. All the morphological parameters were calculated using ImageJ (NIH) except for astrocytic process width which was calculated by SpineJ (Levet et al. 2020, Methods). Ca2+ parameters were calculated by LCPro (Francis et al. 2012, AJP Cell Physiology) and verified using Igor Pro (version 6.36). Statistics were performed using GraphPad PRISM 8.

For manuscripts utilizing custom algorithms or software that are central to the research but not yet described in published literature, software must be made available to editors/reviewers. We strongly encourage code deposition in a community repository (e.g. GitHub). See the Nature Research guidelines for submitting code & software for further information.

Data

Policy information about availability of data

All manuscripts must include a data availability statement. This statement should provide the following information, where applicable:

- Accession codes, unique identifiers, or web links for publicly available datasets
- A list of figures that have associated raw data
- A description of any restrictions on data availability

The data that support the findings of this study are available from the corresponding author upon reasonable request.

Field-specific reporting

Please select the one below that is the best fit for your research. If you are not sure, read the appropriate sections before making your selection.

- [x] Life sciences
- [ ] Behavioural & social sciences
- [ ] Ecological, evolutionary & environmental sciences

For a reference copy of the document with all sections, see nature.com/documents/nr-reporting-summary-flat.pdf

Life sciences study design

All studies must disclose on these points even when the disclosure is negative.

Sample size	We repeated all the reported experiments using at least three independent cultures or animals.<br>No sample size calculation was performed. N numbers are comparable to previously published data from STED structural studies (Tønnesen et al. 2014 Nature Neuroscience, Pfeiffer et al. eLife 2018).
Data exclusions	Data were excluded when the focus shifted significantly for time-lapse data (Fig. 4-8, Fig. S4-6), fluorescence bleached during baseline recording (Fig. 5), or STED stacks did not include complete 3D information of the structure that Ca2+ signals were recorded from (Fig. 7, 8, S5).
Replication	All experimental data were reproduced in at least 3 independent cultures or animals.
Randomization	Data were not explicitly randomized. All pups in the same litter were cultured irrespective of the gender which is inherently random.
Blinding	No blinding was performed. All figures except for Figure 7h-k focuses on structural analysis, therefore blinding did not apply. For Figure 7h-k which shows results from pharmacological experiments, we decided that blinding was not necessary due to descriptive nature of the experiment. Transgenic mouse lines or human patients where identity of the subject can be hidden were not used in the study.

Reporting for specific materials, systems and methods

We require information from authors about some types of materials, experimental systems and methods used in many studies. Here, indicate whether each material, system or method listed is relevant to your study. If you are not sure if a list item applies to your research, read the appropriate section before selecting a response.

Materials & experimental systems

n/a	Involved in the study
[x]	[ ] Antibodies
[x]	[ ] Eukaryotic cell lines
[x]	[ ] Palaeontology
[ ]	[x] Animals and other organisms
[x]	[ ] Human research participants
[x]	[ ] Clinical data

Methods

n/a	Involved in the study
[x]	[ ] ChIP-seq
[x]	[ ] Flow cytometry
[x]	[ ] MRI-based neuroimaging

Animals and other organisms

Policy information about studies involving animals; ARRIVE guidelines recommended for reporting animal research

Laboratory animals	Slices with ZsGreen-labelled astrocytes were obtained by cross-breeding tamoxifen inducible heterozygous GFAP-cre (Tg(GFAP-cre/ERT2)1Fki 41) and homozygous Ai6 (RRID: IMSR_JAX:007906) mice. For slices with YFP-labelled neurons, pups were produced by cross-breeding C57Bl/6J (RRID: IMSR_JAX:000664) with homozygous Thy1-YFP-H (RRID: IMSR_JAX:003782) mice. We used C57Bl/6J wild-type for imaging in organotypic and acute slices as well as in vivo.<br>Mice were housed under a 12 h light/12 h dark cycle at 20 to 22°C with ad libitum access to food and water in the animal facility of the Neurocentre Magendie (Inserm/the University of Bordeaux), and monitored daily by trained staff. All animals used were free of any disease or infection at the time of experiments. Both males and females are used for organotypic slices (cultured at P5-7 and used after 4-5 weeks) or acute slice experiments (8-9 week old). For in vivo experiments, only male mice (8-9 week old) were used.
Wild animals	No wild animals were used.
Field-collected samples	Field-collected samples were not used.
Ethics oversight	Experimental procedures were in accordance with the French National Code of Ethics on Animal Experimentation and approved by the Committee of Ethics of Bordeaux. All procedures were in accordance with the guidelines of the European Directive 2010/63/UE.

Note that full information on the approval of the study protocol must also be provided in the manuscript.
