# Supplementary material for: Structural basis of astrocytic Ca2+ signals at tripartite synapses
Source: Nat Commun. 2020 Apr 20;11:1906. doi: 10.1038/s41467-020-15648-4 (PMC7170846; doi:10.1038/s41467-020-15648-4)
Supplement: Supplementary file 2 — Peer Review [file 41467_2020_15648_MOESM2_ESM.pdf]

Editorial Note: This manuscript has been previously reviewed at another journal that is not operating a transparent peer review scheme. This document only contains reviewer comments and rebuttal letters for versions considered at *Nature Communications*. Mentions of prior referee reports have been redacted.

REVIEWERS' COMMENTS:

Reviewer #1 (Remarks to the Author):

Arizono and colleagues provide a comprehensive supra-resolution STED microscopy analysis of astroglial Ca2+ signals.
So far, the analysis of intracellular Ca2+ signals in living nervous tissue is limited by the temporal and spatial resolution of light microscopy, which is, unfortunately, the only available technique for this purpose. The limitations are particularly evident when Ca2+ signals have to be recorded from astrocytes, one of the major glial cell types. Astrocytes possess filopodial and lammelopodial processes with a thickness frequently ranging from 50 to 400 nm. Many of those are adjacent to synapses where they sense neuronal activity and process this information as a Ca2+ signal.
Here, the authors took advantage of the development of STED microscopy which can provide a spatial resolution well below 100 nm. Using 1P and 2P STED microscopy in 2D and even in 3D, they revealed a high-resolution structure of the spongiform network of astroglial processes. Using multi-channel labeling of cellular structures, extracellular space or free Ca2+, they provide high quality information on the neuronal environment as well as physiological Ca2+ signals in astroglial processes.
Their main observations are:
1. Astrocytes processes possess specialized compartments, called nodes by the authors, that are adjacent to spines. These nodes are highly localized sites of short astroglial Ca2+ signals. FRAP experiments suggest the presence of intracellular boundaries of nodes to other parts of the cell.
2. The Ca2+ signals partially depend on neuronal activity. In addition, they frequently involve liberation from intracellular stores via activation of IP3 receptor.
3. Astroglial process can form ring-like anastomotic connections.
The experiments have been performed at a very high technical level. Although the majority of data has been obtained from organotypic slice cultures, the basic results were confirmed as well in vivo, in the cortex of anaesthetized mice. The results appear very convincing and are carefully discussed. The manuscript is well written.
For each of the observations of this study, there has already been some experimental evidence. None of them is completely novel. However, the present work provides strong confirmation for existing experimental evidence scattered over numerous studies. In addition, the authors provide a convincing experimental framework for future work in this field.
In summary, this is an important piece of work in the field of astrocyte physiology. Simultanepously, it shows the power of current state-of-the art technologies in microscopy and molecular labeling techniques.

Minor points:
The work could be further strengthened by directly demonstrating ER compartments in the astroglial “nodes”, e.g. using NH2-BODIPY which has recently been described as ER-STEDdable dye.
The authors evoked Ca2+ signals via metabotropic glutamate receptor activation. However, noradrenergic or purinergic signaling might be relevant. Are there data on these signaling pathways?

Reviewer #2 (Remarks to the Author):

[Redacted] the authors have satisfactorily addressed my concerns and significantly improved the study. In particular, they have provided experimental proof that the identified astrocyte structural-

functional domains exist not just in organotypic culture preparations, but also in the intact brain, not just in the CA1 hippocampus, but also in other brain regions. In their final revision, they have provided Ca2+ recordings with adequate temporal resolution to identify the correct spatial-temporal sequence of local Ca2+ dynamics and raw Ca2+ movies to support their conclusions (for this reason I suggest that those movies are added also to the present version). Overall, this important piece of work provides an unprecedented level of structural and functional information at the scale of individual synapses and their dialogue with related astrocyte "nodes". The study contains a large amount of novel and precise information. It advances the field of local neuron-astrocyte communication and sets the basis for future work. For these reasons, the study deserves timely publication in Nature Communications.

We thank the reviewers for their positive comments in support of our study (*in italics*). We have addressed the remaining minor points below (in blue).

REVIEWERS' COMMENTS:

Reviewer #1 (Remarks to the Author):

Arizono and colleagues provide a comprehensive supra-resolution STED microscopy analysis of astroglial Ca2+ signals.
So far, the analysis of intracellular Ca2+ signals in living nervous tissue is limited by the temporal and spatial resolution of light microscopy, which is, unfortunately, the only available technique for this purpose. The limitations are particularly evident when Ca2+ signals have to be recorded from astrocytes, one of the major glial cell types. Astrocytes possess filopodial and lammelopodial processes with a thickness frequently ranging from 50 to 400 nm. Many of those are adjacent to synapses where they sense neuronal activity and process this information as a Ca2+ signal.
Here, the authors took advantage of the development of STED microscopy which can provide a spatial resolution well below 100 nm. Using 1P and 2P STED microscopy in 2D and even in 3D, they revealed a high-resolution structure of the spongiform network of astroglial processes. Using multi-channel labeling of cellular structures, extracellular space or free Ca2+, they provide high quality information on the neuronal environment as well as physiological Ca2+ signals in astroglial processes.
Their main observations are:
1. Astrocytes processes possess specialized compartments, called nodes by the authors, that are adjacent to spines. These nodes are highly localized sites of short astroglial Ca2+ signals. FRAP experiments suggest the presence of intracellular boundaries of nodes to other parts of the cell.
2. The Ca2+ signals partially depend on neuronal activity. In addition, they frequently involve liberation from intracellular stores via activation of IP3 receptor.
3. Astroglial process can form ring-like anastomotic connections.
The experiments have been performed at a very high technical level. Although the majority of data has been obtained from organotypic slice cultures, the basic results were confirmed as well in vivo, in the cortex of anaesthetized mice. The results appear very convincing and are carefully discussed. The manuscript is well written.
For each of the observations of this study, there has already been some experimental evidence. None of them is completely novel. However, the present work provides strong confirmation for existing experimental evidence scattered over numerous studies. In addition, the authors provide a convincing experimental framework for future work in this field.
In summary, this is an important piece of work in the field of astrocyte physiology. Simultaneously, it shows the power of current state-of-the art technologies in microscopy and molecular labeling techniques.

We thank Reviewer 1 for his/her positive and thoughtful comments, praising the technical quality of our study and its importance for the field.

Minor points:
The work could be further strengthened by directly demonstrating ER compartments in the astroglial “nodes”, e.g. using NH2-BODIPY which has recently been described as ER-STEDdable dye.

Indeed, a direct demonstration of ER structures in the nodes will be a good next step in the molecular characterization of the nodes. However, several technical hurdles would have first to be overcome to achieve this, necessitating a major new effort. One problem is that organic dyes, such as NH2-BODIPY as suggested by the reviewer, will be taken up by all cells (not just glia but also neurons) in the brain slice, making it very hard / impossible to specifically see astrocytic ER. For this reason, genetic strategies have been developed to label neuronal ER in brain slices by expressing a fluorescent protein tagged with an ER retention sequence under a cell-type specific promotor (Holbro et al. 2009). However, we do not know if the ER retention sequence that works for neuronal ER would also work for fine astrocytic processes where protein sorting mechanisms may be different. In addition, ER labeling inside astrocytic fine processes will likely suffer from substantial bleaching, making STED extremely challenging.

The authors evoked Ca2+ signals via metabotropic glutamate receptor activation. However, noradrenergic or purinergic signaling might be relevant. Are there data on these signaling pathways?

Chances are that nodes are equipped with a plethora of receptors (including noradrenergic and purinergic receptors and their associated signaling complexes) that might modulate the calcium activity we have observed here. However, the focus of our study was to reveal the nanoarchitecture underlying astrocytic calcium signals at tripartite synapses. Identifying other signaling cascades, that might also take place at the nodes, is beyond the scope of the study.

Reviewer #2 (Remarks to the Author):

the authors have satisfactorily addressed my concerns and significantly improved the study. In particular, they have provided experimental proof that the identified astrocyte structural-functional domains exist not just in organotypic culture preparations, but also in the intact brain, not just in the CA1 hippocampus, but also in other brain regions. In their final revision, they have provided Ca2+ recordings with adequate temporal resolution to identify the correct spatial-temporal sequence of local Ca2+ dynamics and raw Ca2+ movies to support their conclusions (for this reason I suggest that those movies are added also to the present version). Overall, this important piece of work provides an unprecedented level of structural and functional information at the scale of individual synapses and their dialogue with related astrocyte "nodes". The study contains a large amount of novel and precise information. It advances the field of local neuron-astrocyte communication and sets the basis for future work. For these reasons, the study deserves timely publication in Nature Communications.

We thank Reviewer 2 for the strong endorsement of our study, commending its technical maturity and importance for the field and recommending its timely publication. We have added the movies as requested.
